# Antimicrobial efficacy, mode of action and in vivo use of hypochlorous acid (HOCl) for prevention or therapeutic support of infections

**DOI:** 10.3205/dgkh000433

**Published:** 2023-03-27

**Authors:** Dirk Boecker, Zhentian Zhang, Roland Breves, Felix Herth, Axel Kramer, Clemens Bulitta

**Affiliations:** 1 TOTO Consulting LLC, San Jose CA, USA; 2 Institute for Medical Statistics, University Medical Center Göttingen, Göttingen, Germany; 3 Henkel AG & Co KGaA, Düsseldorf, Germany; 4 Thoraxklinik, University of Heidelberg, Heidelberg, Germany; 5 Institut of Hygiene and Environmental Medicine, University Medicine Greifswald, Greifswald, Germany; 6 Institut für Medizintechnik, Ostbayerische Technische Hochschule (OTH) Amberg-Weiden, Amberg-Weiden, Germany

**Keywords:** hypochlorous acid, in vivo application, HOCl chemistry, innate immune response, tissue compatibility, infection control, inhalation, wound care, disinfection

## Abstract

The objective is to provide a comprehensive overview of the rapidly developing field of the current state of research on *in vivo* use of hypochlorous acid (HOCl) to aid infection prevention and control, including naso-pharyngeal, alveolar, topical, and systemic HOCl applications. Also, examples are provided of dedicated applications in COVID-19.

A brief background of HOCl’s biological and chemical specifics and its physiological role in the innate immune system is provided to understand the effect of *in vivo* applications in the context of the body’s own physiological defense mechanisms.

## Abstract (de)

Es wird ein umfassender Überblick über den aktuellen Stand der Forschung zum In-vivo-Einsatz von hypochloriger Säure (HOCl) zur Infektionsprävention und -bekämpfung gegeben. Der Schwerpunkt liegt auf nasalen, alveolären und topischen Anwendungen. Außerdem werden Beispiele für spezielle Anwendungen bei COVID-19 vorgestellt.

Um die Wirkung von HOCl im Zusammenwirken mit körpereigenen physiologischen Abwehrmechanismen zu verstehen, werden die biologischen und chemischen Besonderheiten von HOCl und seiner physiologischen Rolle im humanen Immunsystem erläutert

## Introduction

The recent recognition of HOCl in its role as one of the “first responder” agents in the natural defense systems of mammals and most other vertebrates (including fish), creates an exceptional opportunity in the field of infection control in the broadest sense.

The use of HOCl is not new. For practical purposes, the first form of HOCl – called “Eau de Javel” (today in France a synonym for ‘bleach’) – was produced over 200 years ago by Percy and Berthollet in the French village of Javel as potassium hypochlorite (KOCl). Shortly afterwards, but before Pasteur discovered that living organisms can cause infectious diseases, Labarraque and Semmelweis found that HOCl was effective in preventing wound infections and the transmission of puerperal fever [1], [2]. Its anti-infectious properties were recognized even before the widespread use of aqueous chlorine as an antiseptic for traumatic illnesses.

During World War I, the use of HOCl was developed for general hygiene, wounds and therapeutic applications in gangrene, diphtheria, and scarlet fever [2], [3]. Aqueous chlorine has been the antiseptic of choice for several decades. However, the preparation of the solutions, which should be high in HOCl, was difficult and the results varied widely. The instability of many of the solutions produced resulted in rapid degradation over hours or days into several chlorine species, including molecular chlorine (Cl_2_), chlorate (ClO_3_^–^), hypochlorite (ClO^–^), and other molecules that were toxic to tissues and corrosive to surfaces. When solutions mainly consisted of HOCl, they were most effective and had the highest disinfecting power [3].

In the 1940s, aerosolized solutions of acidified hypochlorite were studied quite intensely, and the results led to applications in London hospitals as a control measure against airborne pathogens [4], [5], [6], [7], [8], [9], [10], [11], [12]. During this phase, there were several research groups studying the biocidal benefits of aerosolized HOCl. At that time, a clear understanding already existed of the contribution of HOCl – not the chlorite ion (OCl^–^), which is partially in solution – to the observed result [2], [3]. Decades later it was discovered that HOCl is also naturally formed in activated human neutrophils and other phagocytes belonging to that tissue.

Today, HOCl is used in the areas of health, food safety, water treatment and general hygiene. In the last two decades, more hundreds of reports have appeared describing the use of HOCl (e.g., for disinfection in hospitals, wound antisepsis, extended-care facilities, animal husbandry) [2], [3], [13], [14], [15]. Due to its ability to denature proteins, HOCl can also deactivate prion proteins [16]. All this has opened new ways of combating disease in the healthcare sector.

The use of HOCl in medicine has entered a new phase. HOCl is morphing from a well-accepted disinfection chemical component to an increasingly used *in vivo* agent for applications in wound care and the naso-pharynx. It serves two main roles as:

1. An essential physiological biocidal factor representing one of the first-line defenses against inbound pathogens as the key agent of the innate immune response system and
2. A chemical disinfectant used for a wide variety of industrial and medical disinfection applications [17], [18].

The research on *in vivo* HOCl applications has even extended into prophylactic and therapeutic areas, which was certainly stimulated by the Corona pandemic but is not limited to that field. 

In the scientific community, the *in vivo* use of HOCl is not undisputed because of its complex chemistry and potential side effects. To allow a more informed discussion on the matter within the scientific community, it seemed prudent to provide a comprehensive review of the state of the art of *in vivo* HOCl applications and place it in the context of a solid background of related HOCl chemistry. 

## HOCl – physicochemical properties

### Antimicrobially reactive species

**Figure 1:**
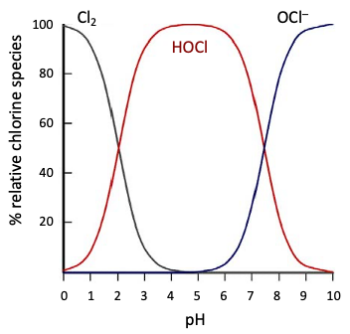
Equilibrium pattern of Cl_2_, HOCl, and OCl^–^ as a function of pH and prevalence of various chlorine species in water [20], [21]

HOCl and its conjugated base OCl^–^ are a potent oxidizing redox system [E0’=+0.9(OCl^–^); E0’=+1.48V(HOCl)] under physiological conditions. The major antimicrobially active species is thought to be HOCl (compared to the hypochlorite anion), consistent with the half-cell oxidation-reduction potentials and an increased ability of the uncharged HOCl species to penetrate bacteria cell walls and membranes of pathogens [19]. Under physiological conditions at a pH of about 7.5, the concentration of HOCl and its correlated base form OCl– are approximately equal (Figure 1 (Fig. 1); [20], [21]).

This potent oxidant system (HOCl/OCl^–^) serves as an endogenous microbicidal agent, generated by myeloid lineage-derived effector cells (including neutrophils). Some high-risk pathogens, like the non-enveloped human papillomaviruses (HPV) – which often contaminate non-autoclavable medical instruments [22] – have shown their highest susceptibility to HOCl [23]. 

**Figure 2:**
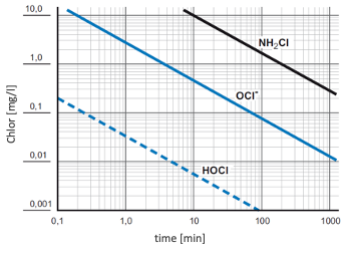
Disinfection efficacy of different chlorine species (HOCl, OCl^–^, NH_2_Cl) for 99% inactivation of *E. coli* (according to [25], [26])

The harmful effect of sodium hypochlorite (NaOCl) on tissue is often presumed to be synonymous with that of HOCl (the same applies to the comparison of Cl_2_ and HOCl). Aqueous solutions of NaOCl – or ‘bleach’ – are strongly alkaline with a pH>9. One must differentiate between the use of aqueous HOCl solutions and the application of NaOCl solutions. The key agent in HOCl solutions at pH levels of 4 to 5.5 is the non-dissociated HOCl molecule, whereas in NaOCl solutions, the active component is OCl^–^. The latter has substantially lower biocidal activity but significant cytotoxic effects [24]. Severing et al. [24] compared the biocidal efficacy of several commercially available NaOCl solutions (pH>8) with conventionally used 0.04% polyhexanide (PHMB) irrigation solution and 0.1% octenidine-dihydrochloride/phenoxyethanol (OCT/PE). Their results demonstrated that low-dosed NaOCl (<0.08%) wound irrigation solutions are significantly less effective than PHMB-based irrigation solution and OCT/PE. They found similar results when investigating a more comprehensive set of commercially available NaOCl solutions [24] (Figure 2 (Fig. 2) according to [25], [26]). 

HOCl has proven to be substantially more effective than bleach (strongly alkaline solutions with the dominance of OCl^–^), yet does not yield development of specific resistance of individual pathogens [27], [28]. A paper by the Swiss Federal Health Office (BAG) reports the relative biocidal effectivity of HOCl, OCl^–^, and NH_2_Cl against *E. coli* (based on data from Butterfield et al. [25], [26].

Such observations with the specifics of HOCl’s biocidal characteristics are increasingly relevant in terms of the growing development of (multi-) resistant pathogens and with a view to its use in animal husbandry.

The use of NaOCl solutions in commercially available prepackaged products is often preferred over aqueous HOCl solutions produced in-situ because of their long-term stability – years (NaOCl) vs months (HOCl). A study by Murashevych et al. [29] with 30 laboratory animals (mature Wistar Han rats) found no negative tissue or health effects after exposing the animals to a single inhalation exposure of NaOCl solution. They concluded: *“The data obtained allow classifying such solution to the 4^th^ (or even 5^th^ – after additional studies) class of toxicity in accordance with Globally Harmonized System of Classification and Labeling of Chemicals”* [29], [30]. The used concentration of 1.7 mg/m^3^ is 340% above the legal limit of HOCl exposure for humans and about 17-fold the concentration in ‘standard’ inhalation experiments with HOCl. Therefore, even small impurities of aqueous HOCl solutions with OCl^–^ (e.g., by slightly increased pH values) are not likely to cause harmful tissue damage. Nevertheless, aerosolized NaOCl solutions are less toxic than Cl_2_-gas [31].

### UV protection

**Figure 3:**
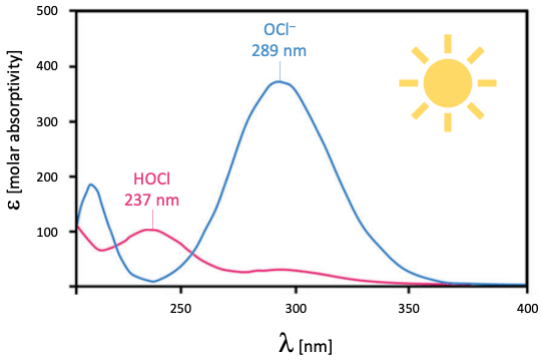
Absorption spectra of HOCl and OCl^–^ (following Snell and Jandova [19])

A more recently identified important physico-chemical feature of HOCl and its corresponding anion [OCl^–^] are their dissimilar UV absorption spectra (Figure 3 (Fig. 3)) [19]. HOCl has its highest absorption at 237 nm and a weaker maximum at 289 nm. Therefore, it can act as a selective filter (like the atmospheric ozone layer) for solar UV[B] photons (280–315 nm) which can damage the skin, while in the process partly undergoing photolysis (UV[A] photons range from 315–400 nm]. 

Such environmental UV[B] and related photolysis reactions induce the formation of various reactive species, including hydroxyl and chlorine free-radicals. It has been postulated that this UV[B] absorption ability might be the basis for HOCl’s potential for protecting against skin cancer [18]. 

Even beyond that, recent studies have explored the interaction between solar UV and HOCl-related environmental co-exposures (e.g., swimming pools), identifying a heretofore unrecognized photo-chemopreventive activity of topical HOCl applications and chlorination stress that blocks tumorigenic inflammatory progression in UV-induced high-risk SKH-1 mouse skin, a finding with potential implications for the prevention of human non-melanoma skin photocarcinogenesis [18], [32].

## HOCl – a natural biocide

### Intracellular HOCl formation 

In biological systems, HOCl can be regarded as the most effective and most common oxidizing agent. It is one of the foremost endogenous molecules for effective phagocytosis of invading bacteria or viruses [33]. The complex control of its physiological creation and the several microbiological functions in extra- and intracellular processes continue make it the subject of a plethora of research projects. 

HOCl is formed as a response to the increase of inflammation markers and immunological stimuli in the body by monocytes and neutrophilic granulocytes (neutrophils in the following) (≈50% of all leukocytes) in the so-called oxidative burst. Here the myeloperoxidase (MPO)-dependent oxidation of chloride anions (using NADPH oxidase-derived superoxide/hydrogen peroxide) produces HOCl and other hypohalous acids as an essential component of the antimicrobial innate immunity [34], [35], [36]. 

The halogenation cycle is located inside the phagolysome of macrophages and microglia cells, where MPO initially reacts with H_2_O_2_ to form an intermediary activated state (compound I) by transitioning its reactive ferric iron (III) site into a highly reactive oxy-ferryl [Fe(IV)=O] radical cation. This ‘activated’ MPO can oxidize intracellular halide ions (e.g., Cl^–^, Br^–^) to form hypohalous acids (e.g., HOCl, HOBr) and regenerate the initial state of MPO with its ferric iron (III) [19].

The net reaction is: H_2_O_2_+H^+^+Cl^–^→ HOCl+H_2_O.

In addition to its role as an intracellular pathogen deactivation compound, HOCl is also extruded through the cell membrane for the purpose of locally oxidizing the ‘attacker’ by various direct reaction schemes.

HOCl is consumed (chlorine is reduced from oxidation level Cl^+1^ to Cl^–1^) and the chloride ion is ‘recycled’. The effectiveness of HOCl produced by neutrophils is impressive: 2x10^–7^ mol HOCl, produced by 10^6^ neutrophils, which can be enough to destroy 150 million *Escherichia coli* bacteria within milliseconds [34]. 

Ulfig and Leichert have presented details of the synthesis and regulation of intracellular HOCl as well as the involvement of the myeloperoxidase reaction schemes in the antimicrobial response and host-pathogen interaction [33]. 

### HOCl – key player of the innate immune response system 

Naturally produced by the innate immune system, HOCl has known antimicrobial properties that also break down biofilm. It has also been shown to be anti-pruritic and anti-inflammatory, as well as to increase oxygenation to wound sites [37]. The physiological functions of HOCl can be categorized according to its localized tasks:

1. Extracellular: Pathogen deactivation and taurine chlorination
2. Intracellular: Pathogen oxidation, inhibition of the maturation of virions, and support of interleukin-6 (Il-6) depletion.

The strength of the current pandemic disease (COVID-19) lay in the fact that the virus was unknown to the human immune system. Persons infected for the first time possess no developed immunity to the pathogen. Nevertheless, when a virus invades our cells, receptors in the cytoplasm recognize the foreign intruder and induce innate immune responses to block replication of the virus. 

It is critical that such innate responses be robust, as it takes three to five days to generate the beginnings of the second line of defense, i.e., the adaptive immune response. The front-line innate defense is often powerful enough to eliminate the virus prior its ability to establish a severe late-phase infection, which relies on adaptive or previously acquired immunity for resolution [38]. 

HOCl is one of the key elements of the body’s innate immune response system, based on one of the highest redox potentials of all physiological intracellularly occurring oxidative, enzyme-controlled defense mechanisms (along with less prevalent hypobromous acid and the lactoperoxidase (LPO) generated hypothiocyanite [OSCN^–^]) [39], [40], [41],[42].

HOCl is highly active against all bacterial, viral, and fungal human pathogens (including spores). It leads to cell death of invading microbes by numerous reaction schemes [43] oxidation of sulfhydryl enzymes and amino acids, ring chlorination of amino acids, loss of intracellular contents, decreased uptake of nutrients, inhibition of protein synthesis, decreased oxygen uptake, oxidation of respiratory components, decreased adenosine triphosphate production, breaks in DNA, and depressed DNA synthesis. HOCl also interacts with structural proteins, such as the capsid or surface compounds, lipid envelope and DNA/RNA materials of viruses [44]. 

Most studies of innate immunity have focused on leukocytes such as neutrophils, macrophages, and natural killer cells. However, epithelial cells play also key roles in innate defenses, including providing a mechanical barrier to microbial entry, signaling to leukocytes, and directly killing pathogens. Importantly, all these defenses are highly inducible by HOCl in response to the sensing of microbial and host products. 

In healthy lungs, the level of innate immune epithelial function is low at baseline. This is indicated by low levels of spontaneous microbial killing and cytokine release, reflecting low constitutive stimulation in the nearly sterile lower respiratory tract when mucociliary clearance mechanisms are functioning effectively. This contrasts with the colon, where bacteria are continuously present and epithelial cells are constitutively activated. Although the surface area of the lungs presents a large target for microbial invasion, activated lung epithelial cells that are closely opposed to deposited pathogens are ideally positioned for microbial killing via HOCl-induced mechanisms [45]. 

There is a high likelihood that HOCl rapidly modifies a variety of constituents of both extracellular and intracellular fluids. The interaction with taurine (H0_3_S-CH_2_-CH_2_-NH_2_) is one of the most prominent, resulting in the formation of N-chlorotaurine (NCT) in the extracellular medium. In the presence of hydrogen peroxide and chloride, MPO catalyzes the formation of HOCl, which reacts with amino acids, forming chloramines [46]. In this way, the oxidizing power of HOCl is modulated by the formation of the weaker oxidant NCT. This is transported to the intracellular medium and there reduced again to taurine, where it serves as a precursor for several metabolic functions and as a cell-membrane stabilizing component [47]. Taurine is present in body fluids in amounts that can reach 0.1% of total body weight and is particularly likely to be involved in the biological effects of HOCl exposure. HOCl and NCT have anti-inflammatory properties which lead to reduced local inflammation, lower infectivity through reduced permeability, and perhaps lower morbidity and mortality overall [48], [49]. The verified unique stability and low-level reactivity of NCT are considered essential for both its function in the mammalian defense system and its practical applicability, which manifests itself in an optimal compromise between microbiocidal activity and toxicity [50], [51]. 

HOCl has been suggested to have a role as an anti-inflammatory agent beyond its direct antiviral efficacy. By inactivating interleukin 6 (IL6), HOCl possibly downregulates mediators of the ‘cytokine storm’ that characterizes clinical deterioration in specific diseases (e.g., COVID-19) [48], [52], [53]. Based on their *in vitro* studies on the deactivation of IL-6 by HOCl at concentrations reported to develop *in vivo*, Robins et al. [54] suggest that exogenous HOCl and NCT might bring about beneficial effects in the treatment of COVID-19. Their results and further studies on how HOCl and HOBr and their halogenated amine derivates interact with IL-6 may open alternative therapeutic interventions in COVID-19 and other hyperinflammatory diseases.

### Defense against airborne infection processes 

Airborne pathogens are created during speaking, coughing, sneezing, or spitting as droplets or nuclei. Larger exhaled droplets sink to the floor, while light-weight aerosol particles (<10 µm) can remain airborne for many hours. Airborne and surficial pathogens can remain stable and contagious for hours or days and present a long-term infection risk [55]. Galvin et al. [56] recommend HOCl for disinfecting offices, with an emphasis on its disinfection use for dental clinics. However, evidence of reduced morbidity is lacking. They also stress the general importance of fog- or mist-based disinfection in non-populated areas, given the aerosol property of the exhaled virus laden particles [44].

Depending on their size, floating aerosols are ‘filtered’ by the upper airways, while the smaller aerosols (<5 µm) may reach the tracheal and lower respiratory tract [57], [58]. The susceptibility for infections of the lower lung areas arises from its gas-exchange architectural requirements. To promote ventilation, about 100 m^2^ surface of the lung are continuously exposed to satisfy the gas diffusion demand into a thin capillary tissue [59]. Every minute, 5 to 10 L of ambient air is vented, which may include particles, droplets, and pathogens. The lung has a large environmental interface with only minimal defense barriers to its alveoli. Despite this structural permeability, the lungs successfully defend against most infectious challenges through a variety of mechanisms, such as mucus layer, mucociliary mechanics, antibody and antimicrobial peptides, alveolar macrophages, and leukocyte recruitment promoted by the pulmonary epithelium [59], [60]. When basal defenses are overcome, the pulmonary epithelium responds by increasing its direct antimicrobial capabilities and directing leukocyte recruitment from the circulation [61]. Just as the accessibility and large surface area of the lungs contribute to infectious susceptibility, these features also provide a unique opportunity for topical therapy in aerosol respiratory form [62].

The clinical symptoms of an airborne infection, explained using COVID-19 as an example, can be divided into three phases (Table 1 (Tab. 1)) [63].

### Incubation phase

Infections spread by airborne pathogens follow certain stages with respect to its successive progression of effected anatomical structures and areas. The infection process begins with inhaling the pathogen and its establishment in the respiratory epithelium via various ‘entry ports’, such as the mucosa in the mouth, nose, throat, trachea, upper and lower alveolar spaces, ocular surface, or indirectly, through skin and fomites contaminated by these droplets and auto inoculated into the eyes, nose, or mouth [59], [64]. It is important to note that the nasal epithelia are not only a critical entry port for but also a major exit point of pathogens (i.e., source when exhaling). The second target in the early infection process the epithelial cells of the salivary glands, which also can become a significant source of pathogens particularly in the early phase of infection by emitting saliva droplets [65].

Once the mucus barrier is overcome, further implantation of the virus or bacteria follows via direct contact and bonding to the mucosal epithelia. The attachment for most Gram-negative bacteria occurs via pili (fimbriae) – thin, hair-like protein appendages on their surface – that bind to specific membrane factors on the host cells. Similarly, viruses use specific protein domains to establish a connection with certain receptors of the host cells; further on, they enter the cytoplasm (by endocytosis), access the cell’s synthetic apparatus, and use it for their own replication. The virulence factors expressed by the pathogen to serve for its attachment to the host cells are termed “adhesins”. The role of such adhesins for all Corona viruses is played by the surface glycoprotein spike (S-protein) and its receptor-binding domain [66].

The viral load in the pharynx is high during the first week, when the symptoms are moderate or prodromal, with a peak on day 4, which suggests active replication in the upper respiratory tract [67], [68]. The hACE2 receptor, used by SARS-CoV-2 to establish infection, is highly expressed in the mucosa of the oral cavity and nasal tissues [68], [69], [70], [71].

### Acute phase 

The next phase is started by the virus entering the peripheral blood from the lungs, causing viremia and subsequently attacks of targeted ACE2-expressing organs such as lungs, heart, kidneys, and the gastrointestinal tract [72]. 

### Recovery phase 

The recovery phase can take two routes. Full recovery is gained in most cases within a few weeks. In a small percentage of cases, recovery takes much longer, i.e., long flu and long COVID; the latter is still being studied intensively [73].

## Safety – HOCl tissue compatibility and tolerance

### Cytotoxicity

To test the upper limit of safe exposure, human cells can be stress tested with HOCl solutions of different concentrations to determine the limits of their physiological resilience. There are numerous works on this in the more recent literature [3], [33], [34], [35], [74], [75], [76], [77]. Further resources can be found in toxicology databases [78]. On eyes, skin and wounds of rats and pigs, HOCl concentrations of 0.01% to 0.1% (i.e., 100 ppm–1,000 ppm) were tolerable [35]. In humans, cells cultured from the umbilical vein, showed that HOCl was tissue-compatible up to 1,300 ppm [79]. For aqueous NaOCl/HOCl solutions, no skin irritation whatsoever was found at 250 ppm (0.025%) [16], [80]. 250 ppm proved to be bactericidal and at the same time tissue-friendly (*in vivo* human skin incisional model). With more concentrated solutions of 2,500 ppm (0.25%), weak tissue irritations were observed [81]. Stabilized HOCl solutions proved to be non-irritating (spray of 130 ppm HOCl solution into the ocular skin of rabbit eyes) and non-sensitizing in dermal studies (1,000 ppm HOCl solution) on guinea pigs [44]. A 28-day dermal application study (Guinea pigs) with a 1,000 ppm HOCl solution showed no evidence of systemic toxicity [35], [44]. 

Fidler et al. studied possible virus transmission using ultrasonic scalers (USS) in periodontal treatment, and its prevention by adding HOCl as an antiviral agent injected into the cooling fluid during operation. In their studies, HOCl was compared to NaOCl. It proved not to be cytotoxic (unlike NaOCl) and had a superior biocidal effect [28]. 

HOCl solutions with their proven antimicrobial efficacy were found to be safe for skin and eye according to *in vitro* biocompatibility studies [82]. While the antimicrobial actions of regularly used disinfectants are well understood, much less is known regarding their dermatological reactions, including but not limited to irritation and hypersensitivity [83]. For HOCl solutions, however, at concentrations at or below approved levels, there are no reports of adverse reactions to topical applications, either from the Toxicology Database DSSTox of Environmental Protection Agency (USA) [84], the US CDC Toxic Substances and Disease Registry [85], the Development and Reproductive Toxicology Database, or the European Bioinformatics Institute of EMBL [21]. 

### Inhalative toxicity 

In the gas phase, the interaction of HOCl molecules with tissue cells is diffusion controlled. The semi-quantitative estimate of cell exposure caused by the suggested atmospheric concentration of 0.25 ml/m^3^ of a 500-ppm HOCl solution (computational framework: exposure 8 hours, all available HOCl reacting with the tissue, breathing volume 0.5l, exposure time 5 min; comparable to the test on cells with HOCl solution [76]) is equivalent to the load of an aqueous HOCl solution of 6.9x10^–5^ mmol/l for a tissue sample. The value of 6.9x10^–5^ mmol/l is significantly below the legal limit [86] for harmful substances of 0.5 mg/m^3^ (corresponding to the effective equivalent exposure of 4.6x10^–4^ mmol/l) and 4 orders of magnitude below the TC50 value, measured with aqueous HOCl solutions (3.3x10^–1^ mmol/L) [76]. 

Recent studies confirmed the fast reduction (log 2 in less than 1 minute) of microbes (*E. coli*) by an HOCl atmosphere (atomization of 500 ppm Biodyozon^®^; 3ml/m^3^) [87]. Also, the toxicity test of the HOCl medium was passed (HOCl solution, 200 ppm) and the bactericidal effect of aerosolized HOCl on culture plates was successfully validated [88]. Several studies against a range of bacteria confirmed high effectivity already at 10–50% of the regulated atmospheric limit for HOCl concentrations [89], [90]. 

In animal studies with massive virucidal vapor exposure far in excess of the minimum necessary to be effective as a virucidal agent (8h/d, 2 weeks, 250 ppm HOCl fog), no detectable blood parameter change, nor any significant change of lung function was observed [91]. Also, in human studies no observable changes in the endoscopic scores or any adverse effects were detected during regular exposure with HOCl via nasal irrigation (twice daily, 30 ml, 3.5 ppm HOCl solution for 8 weeks) [92], [93]. The studies show that with a protective, HOCl-laden atmosphere of a concentration <0.21 ppm (0.5 mg/m^3^), no tissue irritation is to be expected. Aerosolized HOCl in this concentration is considered harmless to humans [3]. Considering the similar susceptibility of enveloped viruses and bacteria in suspension tests [90], [94] an efficacy against enveloped viruses (including Corona viruses) could be concluded. 

### Disinfecting by-products 

The use of active chlorine components like HOCl is not undisputed. A significant body of research reports that the principal biochemical reaction mechanisms of HOCl with biological structures and organic substrates potentially produces harmful effects.

An area of HOCl-related concern is the negative effect of disinfecting by-products (DBP) in the water and atmosphere of (public) swimming pools [95], [96]. Several studies report respiratory problems in recreational and professional swimmers [95], [97], [98], [99], [100], [101]. The actual substances which appear to cause the damaging tissue effects are the DBPs, namely trihalomethanes (THM), nitrogen-containing products, like haloacetonitriles, nitrosamines, and (mono, di, tri)-chloramines, which have been identified among another 100 different DBPs [96], [102], [103], [104], [105], [106], [107]. 

The mutagenicity of the investigated pool waters resembled that of drinking water. Subjects who swam in the mutagenic, chlorinated pool water evaluated had increases in genotoxicity biomarkers that were associated with the concentration of brominated THMs [96]. Remarkably, in none of the reviewed studies listed here, HOCl or active chlorine was measured in the pool’s atmosphere. Nevertheless, some of the studies attribute the negative effect to HOCl [108], which the present authors could not verify. This is important when considering vaporized HOCl for air-disinfection purposes, because in that case, the creation of pool-like DBPs is not to be expected. 

### Chlorination stress and mutagenicity 

Most of HOCl related adverse reactions can be grouped under the term ‘chlorination stress’ as defining the sum of pathological interaction in a physiological organism caused by HOCl or its chemical ‘derivatives’, that is, breakdown products often observed in connection with and linked to the process of ‘oxidative stress’ and its counter measure of ‘antioxidants’. Unfortunately, the use of aerosolized HOCl is often viewed as equivalent to direct exposure with HOCl-related compounds, such as chlorine gas (Cl_2_) and OCl^—^ (from dissociation of NaOCl [bleach]) or other commonly used disinfectants, e.g. hydrogen peroxide (H_2_O_2_), and ozone (O_3_).

Chlorination stress that occurs under physiological conditions or due to environmental exposure influences the structure and function of numerous classes of biomolecules, either through covalent introduction of chlorine (and chlorine-derived substituents) or through indirect oxidative insult (‘oxidative stress’). Chlorination is mainly linked to diseases where molecular alterations constitute the major suspected cause: i.e., inflammation, tissue lesions, DNA damage, apoptosis, and oxidative stress itself [109].

HOCl reacts with a variety of organic matter, usually creating intermediate radicals which themselves – in a cascading reaction – ultimately produce smaller hydrocarbons [110]. HOCl-dependent chlorination stress, caused by an exceedingly high and out-of-control endogenous HOCl concentration – is dictated by both thermodynamic and kinetic parameters that ultimately determine the susceptibility of various biochemical targets, e.g., nucleotides, N-halo compounds, sulfurous amino acids, protein side-chains, peptides, and lipids [19], [110], [111], [112], [113], [114]. 

Several of the diseases induced by this stress either promote or provoke age-related diseases (Table 2 (Tab. 2)). Although HOCl serves innate host-defense functions, it may also induce tissue damage at sites of inflammation, an area of active research in neurodegenerative disease (e.g., Alzheimer’s or Parkinson’s disease), metabolic and cardiovascular dysfunction (atherosclerosis; diabetes), autoimmune dysregulation, asthma, cancer, and chronological aging, among others [19], [108], [115], [116]. 

The generation of HOCl has been demonstrated to interfere with lipoproteins, with the consequent oxidation resulting in the conversion of macrophages into foam cells [type of macrophages localized in fatty deposits on blood vessel walls, where they ingest low-density lipoproteins and become laden with lipids, giving them a foamy appearance] and in the acceleration of the atherosclerotic process. HOCl also induces endothelial dysfunction by interfering with the NO synthetic pathway [108]. In equilibrium at physiological pH with its anionic form (OCl^–^), HOCl may also induce tissue damage at sites of inflammation involving the oxidation and chlorination of biomolecules targeting peptides, proteins, lipids, and nucleic acids [108].

Several reviews and other articles warn against the potential health risks and tissue damage caused by exposure to HOCl. They base their position on the many potentially harmful reaction pathways of the complex chemistry of halous acids in biological systems and tissue (e.g., intermediary products such radicals, reaction cascades of short-lived highly reactive components, cascades of hardly controllable reaction products with a multitude of reaction schemes). Also, they draw parallels to H_2_O_2_ reactions with biological systems and assume similar harmful effects of HOCl itself [117], [118]. 

It is recognized that HOCl can kill pathogens through its strong oxidizing potential. This cytotoxic oxidant also induces significant increases in cellular damage, as reflected in lipid peroxidation, protein oxidation, and DNA damage. Many chronic inflammatory conditions are associated with an increased risk of cancer development. 

Testing the oxidation potential and mutagenicity of NaOCl has a long history, due its use as the main disinfectant for drinking water [119], [120], [121]. As early as 1997, Soffritti et al. reported the results of long-term carcinogenicity bioassays (104 weeks) on chlorine (in the form of sodium hypochlorite and monochloramine in increasing doses from 0, 70, 140, to 275 ppm), administered in drinking water to rats and mice. The incidence of leukemia was higher for the middle (37%) and high dose (32%) compared to the control group (16%) [119]. Kurokawa et al. found no evidence of carcinogenic effects on organs (e.g., liver, pancreas, lungs) from exposure to NaOCl solutions (500 and 1,000 ppm NaOCl solution for drinking water used for 103 weeks), whereas KBrO_3_ showed a clear carcinogenic effect [120].

Ishidate et al. investigated the mutagenicity of NaOCl among their test array of 242 food additives. NaOCl and 10 other food additives tested positive both in the Ames reverse mutation assay (pH 7.4) and the chromosomal aberration test. Sassa et al. studied the effect of HOCl on inflamed tissues [122]. They stated that at the site of inflammation, cellular DNA was damaged by HOCl. 8-Chloro-2’-deoxyguanosine (8-Cl-dG) is a major DNA adduct formed by HOCl and has been detected in the liver DNA and urine of rats given lipopolysaccharide in an inflammation model. Sassa et al. concluded that the HOCl-induced 8-Cl-dG adduct may generate mutations at sites of inflammation [122]. It must be noted that they attributed the mutagenic effect to HOCl, even though they used a 250 mM sodium phosphate buffered solution (pH 8.0) of a 1 mM NaOCl solution. Under these conditions, the key oxidizing component is the hypochlorite ion OCl^–^ (bleach) [20].

While many such principal reaction schemes have been validated in lab experiments, they lack an analysis of the actual effect of the described harmful components at way lower – physiological – concentration levels. This concentration difference presumably explains the measured harmful effects compared to favorable results in clinical studies. For example, vaporized components from bleach solutions (NaOCl_aq_) as residue from surface cleaning [123] procedures only reach concentrations in the ppb range [124]. Exactly which concentrations are potentially harmful remains to be determined (e.g., vaporized HOCl is ‘cleared’ by the European Chemical Agency up to 0.22 ppm (0.5mg/m^3^). It is also yet to be determined whether short-lived components downhill of halous acids reaction cascades (e.g., Cl● or OH● radicals) measurably clinically affect health when occurring at very low concentrations. Furthermore, many of these publications from environmental chemistry refer to NaOCl as interchangeable with HOCl, which does not reflect the distinct effects of the two substances [77]. While NaOCl has demonstrated to be an extremely harmful substance to living tissue [28], [77], vaporized molecular HOCl has not shown any adverse effects at the permitted concentration levels. 

Notably, potential resources and resistance of human cell membranes are higher than microbial membrane resistance. Thus, enzymatic systems in cells and tissues remain intact despite HOCl use. Again, the use of NaOCl (bleach) and the resulting OCl^–^ (e.g., applied via direct fogging/aerosolization) must be differentiated from the use of molecular HOCl per se (e.g., via vaporization). While the former has severely deleterious effects on tissue and biological substrates [77], [125], HOCl itself is much more tolerable and subject to physiological auto-controlling mechanisms in case of overdose. 

### Defense mechanisms against excess HOCl 

Under healthy physiological conditions the living tissue possesses HOCl deactivation processes to keep the overall amount of circulating or present HOCl in check. Given tissue inflammation, however, the HOCl attack on somatic tissue is intensified because of anti-oxidative depression [34], [126]. In such situations, endogenous cells have only a limited enzymatic ability to deactivate HOCl. The ‘target’ germs are destroyed, but an excess of HOCl may lead to irritation of the living tissue and unreasonable impairment [127], [128]. The major antimicrobial products of neutrophilic MPO in physiological fluids are HOCl and hypothiocyanite (OSCN^–^); the former is generally believed to be the killing agent. However, Ashby et al. determined that HOCl oxidizes SCN^–^ in a facile nonenzymic reaction. In that study, the kinetics and computational models substantiate the hypothesis that SCN^–^ serves to moderate the potential autotoxicity of HOCl by restricting its lifetime in physiological fluids. Furthermore, the oxidizing equivalents of HOCl are preserved in OSCN^–^, a more discriminating biocide that is not lethal to mammalian cells [39].

Numerous molecular entities of endogenous or phytochemical origin have been shown to antagonize chlorination stress that occurs due to exposure to HOCl, including amino-acid derivatives (taurine, glutathione, serotonin, carnosine, ovothiol, ergothioneine), phenolics (gallic acid, nordihydroguaiaretic acid, quercetin), and B_6_ vitamers (pyridoxal, pyridoxine, pyridoxamine), attributed mostly to chemical reactivity (i.e., sacrificial quenching). In addition, antagonists of MPO’s enzymatic activity (such as the synthetic MPO inhibitor Verdiperstat or the endogenous metabolite uric acid) blocking HOCl formation have been explored for pharmacological control of pathophysiological chlorination stress [19].

In 2018, the US Food and Drug Administration (FDA) approved the use of products containing hypochlorous acid for a range of medical indications, including use as an antipruritic, cleansing of eyelids, as well as wound cleansing and debridement [129]. The FDA has approved the use of HOCl not only for periocular skin treatment and dental spray application, but also as a no-rinse sanitizer for fruit and vegetables [44], [130].

## In vivo applications of HOCl

### Overview 

The study of *in vivo* HOCl applications has recently seen a renaissance. The development of products to both simulate and augment the physiological HOCl functions has gained enormous interest. In particular, the extracellular attack of pathogens prior to their intracellular replication has become an active area. Externally applied HOCl plays an important role in stimulating a part of the innate immune system known as mucosal immunity. That is a first line of defense targeting the cells lining the respiratory tract and other surfaces such as the intestines and the urogenital tract, which do not tend to be activated by conventional vaccines. A recent study by Agafonova et al. [131] emphasized the importance and fragility of this pathogen defense mechanism. They investigated the alteration of mucosal immunity after infection with COVID-19. They discovered a virus induced immunosuppression which led to functional and metabolic impairment of neutrophils of the mucosal immunity system in recovered COVID-19 patients. Their findings underline the importance of taking measures to protect and strengthen the innate immune response system, for instance using adjunct HOCl-enabled procedures.

All practical ways of administering HOCl have been investigated, demonstrating a safe and effective means of boosting the innate immune response [132]. The methods span nasal and pharyngeal inhalation, topical applications (e.g., wounds), intra-operative (e.g., peritoneal lavage), gastro-intestinal, ocular [130], and even systemic intravenous delivery (e.g., rheumatoid arthritis, supplement in hemodialysis of end-stage renal disease) [133], [134]. 

Externally applied HOCl can play an important role in the protection of respiratory-tract epithelia against viral infections. The importance of an intracellular innate immune response occurring in parallel has also been demonstrated: inhibition of coronavirus infection in cultured epithelial cell lines with externally applied HOCl coincides with intracellular production of HOCl [48]. Apparently, administered HOCl can foster and complement the intracellular HOCl response with direct extracellular pathogen attacks or by stimulating the intracellular HOCl production rate. The connection between HOCl’s extra- and intracellular effects was shown by markedly inhibited rhinovirus replication in primary cultured nasal epithelial cells in which infected cultures had been exposed to HOCl, suggesting that exogenous sources of HOCl may be able to intervene in the maturation of intracellular virions [38], [48].

### Hypertonic saline nasal irrigation and gargle 

A recent scientific discovery demonstrates a natural, safe, and accessible self-administered procedure that enhances our innate immunity. Non-myeloid epithelial cells can produce HOCl. The amount of HOCl generated is dependent on the concentration of available intracellular chloride. *In vitro*, saline exposure of epithelial cells infected with coronavirus significantly reduced viral replication [38].

*In vivo*, hypertonic saline nasal irrigation and gargle (HSNIG) in patients with upper respiratory infections (URI) caused by coronavirus significantly reduced the duration and severity of the infection compared to similarly infected patients treated with the standard of care. HSNIG can suppress replication of SARS-CoV-2, mitigating the spread during the asymptomatic phase and reducing the risk of an asymptomatic or symptomatic case progression to a severe infection [38]. Saline irrigation can be seen as a promoter of the innate HOCl production.

Antiviral activity against a broad range of viral infections can be augmented by increasing availability of NaCl [36]. Using MPO, phagocytes destroy ingested microbes by producing HOCl from Cl^–^ and H_2_O_2_ within phagolysosomes. This suggests that non-myeloid cells possess an innate antiviral mechanism dependent on the availability of Cl^–^ to produce HOCl [36]. 

Many studies evaluating the efficacy of saline irrigation have indicated a clear improvement in the quality of life of patients undergoing treatment for various diseases, including rhinosinusitis and allergic rhinitis, as well as in the postoperative care of patients who have undergone endoscopic sinus surgery [135], [136]. However, saline irrigation should be less effective than HOCl-activated solutions for uncontrolled rhinosinusitis, because saline irrigation by itself lacks an antibacterial effect and mucin is hydrophobic [92], [93], [137], [138].

In addition to nasal irrigation with hypertonic saline solutions, the inhaling approach has been studied extensively. The most important practical advantage of this method is its simplicity and very low probability of adverse effects.

A meta-analysis by House et al. (29 studies, 1,583 patients) on the use of nebulized normal saline over placebo for treatment of acute viral bronchiolitis showed clear benefits. Patients with bronchiolitis treated with nebulized normal saline showed significant improvement in respiratory rate and respiratory score after treatment, although oxygen saturation remained unchanged. Compared with patients treated with other placebos, those treated with nebulized normal saline showed greater improvements in post-treatment respiratory scores [139]. 

### Biofilms 

Brindle et al. showed promising use of HOCl (0.025%), which was the only biocidal agent to successfully eradicate Ralstonia Pickettii in planktonic and mature biofilm on three types of silicone implants. The HOCl solution was shown to be more effective than a triple antibiotic solution (1 g Cefazolin, 80 mg Gentamicin and 50,000 U of Bacitracin in 500 mL saline), 0.05% chlorhexidine digluconate, and 10% povidone-iodine in the penetration and eradication of Ralstonia Pickettii biofilm from the surface of two different types of textured and one type of smooth breast implants *in vitro* [140].

Based on an *in vitro* study, where HOCl solutions (0.025%) demonstrated rapid and broad antimicrobial activity against planktonic and biofilm phenotype bacteria, Haws et al. quantified the antimicrobial ability of HOCl to deactivate reactive proteins in surgical pocket irrigation for breast implants [141]. A semi-quantitative calculation of the ratio of HOCl molecules to protein molecules showed a necessary ratio of 2–3*103 [141].

### Pre-operative and surgical site antisepsis 

Pre- and post-operative management of surgical wounds is important to prevent infection and dehiscence, minimize scar formation, and reduce the risk of other complications (e.g., itching, stiffness, scar contracture, tenderness, and pain, in addition to psychosocial effects caused by scarring)[37]. HOCl solutions (0.01%) have been successfully used for skin and eyelid antisepsis before ocular surgery. The studies showed that the HOCl application significantly reduced the bacterial load without altering the diversity of bacterial species remaining on the skin under the lower eyelid, thus obviating the problem of resistance of certain bacterial strains to various antibiotics [130].

Finger et al. [142] recommended HOCl solutions to assist antiviral pre-examination prophylaxis in ophthalmology, both for patients AND the examiner. They used a 0.01% HOCl spray solution (pH 6.5–7.0) free from sodium hypochlorite. They found it significantly improved patient comfort without showing any short- or long-term complications, preferring it to the common presurgical antibiotic prophylaxis of a 5% providone-iodine solution (betadine). They recommended this procedure as a superior alternative to providone-iodine (betadine) solutions, although epidemiological evidence of prevention of endophthalmitis in contrast to PVP-iodine is lacking to date.

Its use in the preparation of surgical sites helps decrease pathogen loads and therefore the risk of surgical site infections. Rinses are non-irritating and are marketed in easily applied formulas [129]. Block and Rowan reviewed surgeons’ needs for antisepsis during the coronavirus pandemic and concluded that ‘HOCl comprises many of the desired effects of the ideal antiseptic or disinfectant: it is easy to use, is inexpensive, has a good safety profile, and can be used to disinfect large areas quickly and with a broad range of bactericidal and virucidal effects’ [143]. Gold et al. [37] recommended that use of topical HOCl formulations be “strongly considered as a post-surgical/procedural wound healing agent.”

### Managing acute wounds 

HOCl-soaked wound dressings are relied upon for continued delivery of the antimicrobial agent and healing enhancement over time [21], [144]. Cavity wounds that cannot be cleaned using standard irrigating solution are challenging. Successful application of HOCl was demonstrated in treating a horseshoe perianal abscess. The treatment was superior to the standard process of using povidone-iodine with wet-to-dry dressing [145]. Matthews et al. repeatedly instilled an HOCl solution (0.01 %) into the abdomen, which was temporarily closed. They demonstrated a visually decreased contaminated effluent in the intra-abdominal fluid and a decreased abdominal mucopurulence – an effect which they assigned to HOCl clearing the septic abdomen of opportunistic microorganisms [35], [146], [147]. 

### Supportive treatment of venous leg ulcers (VLUs) 

Numerous examples exist of how the use of HOCl has outperformed many conventional (e.g., antibiotic) treatment schemes in terms of efficiency and long-term patient acceptance [148], [149]. In VLUs, compression is the mainstay of treatment. There is observational evidence supporting the use of HOCl as a topical wound solution on VLU, and a randomized controlled trial is underway to determine if such use of HOCl as an effective antiseptic and revitalizing agent can be encouraged [150].

Bongiovanni et al. [151] performed a retrospective analysis of 897 patients with 1,249 venous leg ulcers. They concluded that VLU-care protocols that clean, debride, pack and dress with HOCl solutions can reduce the effects of some co-morbidities while accelerating healing times. They also provided a comprehensive review of use of HOCl in the treatment of more than 1,000 VLU, summarizing as follows [151] “Perhaps the greatest advance in VLU care is the addition of HOCl to the treatment armamentarium. Aqueous solutions of HOCl, even in trace amounts, will kill most pathogens within 30 seconds of exposure. Additional actions of HOCl include reduction of mast cell degranulation and active capillary dilation. The latter effect is of great importance in the diabetic VLU patient since one of the paradoxes in diabetes is the reduction of capillary perfusion via arteriovenous shunting at the microcirculatory level.” Gold et al. [149] review the work of other authors who confirm the clinical efficacy of HOCl solutions. HOCl is often applied as an adjunctive therapy for refractory VLU, which appreciably increases healing and rapidly relieves pain [152]. 

### Chronic wound care 

Although the precise mechanism of wound healing is not fully understood, 3 interrelated phases – inflammation, migration, and remodeling – require coordinated activity for successful wound healing: a progressive series of events facilitated by platelets, leukocytes, fibroblasts, and keratinocytes. Platelets facilitate homeostasis and the release of growth factors, then leukocytes participate in the inflammatory process, fibroblasts break down the fibrin clot, create new extracellular matrix and collagen structure, and finally keratinocytes help cover dermal and mucosal wound surfaces to reestablish an epithelial barrier with the external environment [43], [153]. Wound care methods vary in terms of how they interact with the different stages and related mechanisms. HOCl is known to impact all three phases of wound healing at the cellular level, exhibiting anti-inflammatory, anti-infective, cell proliferative, and immunomodulatory properties, as shown in multiple laboratory analyses [43], [154]. 

Solutions of NaOCl (Dakin’s solution) have been used in wound care for more than 100 years. In the last 15 years, safer HOCl acid solutions, produced using electrochemical techniques, have emerged as safe and viable wound-cleansing agents and adjunct therapy in infection treatment. Such solutions (or stabilized solutions with silicone) show advantages over conventional means in particularly difficult-to-treat cases and chronic wound care. 

Numerous cleansing and topical antimicrobial agents are known to be toxic to many of the cells involved in the wound-healing cascade. Topical antiseptics with a long history of use, such as NaOCI, H_2_O_2_, acetic acid, and povidone-iodine, remain in widespread use today. When used at typical concentrations, these antimicrobial agents are cytotoxic and impede wound healing; hence, their use on chronic ulcers is now generally discouraged by experts [148]. In contrast, HOCI has no cellular toxicity when used in clinically effective dosages. It has been widely reported that the human body regulates the levels of HOCI during the inflammatory response using its own ‘antioxidant-defense’ system based on scavenger molecules, such as taurine and nitrites, which diminish or neutralize HOCI and protect against oxidative damage to cells [155], [156].

HOCl’s positive characteristics appear to correlate with potential therapeutic benefits of topically applied HOCl for antisepsis, wound decontamination, and a variety of skin disorders and healing applications [157], [158], [159]. Treatments are often repeated during the day in the early stages of wound management, adjunctive to debridement and other procedures aimed at adherent soil and biofilm removal [149]. An example of its wide-spread successful use in topical applications has been reported by Fukuyama et al. [49]. They showed that topical HOCl formulations relieve itching in human patients with atopic dermatitis, and studied the specifics of the underlying antipruritic mechanism in a mouse model. They found a direct reduction in sensory response by HOCl, leading to significantly reduced itch and inflammation *in vivo*. The reduced inflammatory response by HOCl treatment manifested as reduced secretion of inflammatory cytokines in affected skin tissue.

Armstrong et al. [160] formulated the recommendations of an expert panel for the use of HOCl in wound care, based on strong and moderate evidence (e.g., diabetic foot wounds, septic surgical wounds). The panel also recommended intralesional use of HOCl for 15 min after debridement. A review of topical HOCl applications is presented by Gold et al. [149]. The latter found that topical stabilized HOCl provides an optimal wound healing environment and, when combined with silicone, may be ideal for reducing scarring. Additionally, in contrast to chlorhexidine, HOCl used as an antiseptic skin preparation raises no concerns of ocular or ototoxicity. For wound care and scar management, topical stabilized HOCl conveys powerful microbicidal and antibiofilm properties, in addition to potency as a topical wound healing agent [149]. 

Biofilms present the main cause for delayed chronic wound healing. Biofilm formation is thought to create a self-perpetuating cycle, prolonging the existence of macrophages and neutrophils in the wound, which in turn impairs normal wound healing and potentially reduces the effectiveness of innate immunological responses [149]. Treatment with HOCl affects biofilm by modifying its structure and therefore facilitating access of HOCl itself or other bactericides to local bacteria, with consequent potential clinical benefits in the treatment of chronic wounds [161]. Studies by Sakarya [43] suggest HOCl solution as an ideal wound care solution with a powerful and rapid killing effect on different types of microorganisms, anti-biofilms, and microbiocidal effect within the biofilm

### Nasal and pharyngeal applications 

Disease severity, progression, and mortality rates caused by airborne pathogens (e.g., SARS-CoV-2) are directly related to the level of viral load, which is formed and determined early in the infection process in the upper respiratory tract [68], [162]. It makes this area a first target for externally applied HOCl treatment of infected and even pre-symptomatic patients (e.g., via nasopharyngeal [nose and throat] spraying and inhaling).

Nasopharyngeal spraying or inhaling micro-aerosolized or vaporized HOCl delivers two beneficial effects:

1. They attack and deactivate the virus either mid-air or in the early process of linking to and reacting with specific cell-wall receptors.
2. They activate the intrinsic intracellular immune response by increasing the MPO-driven HOCl production or suppression of the interleukin6 storm often observed in early phases of infection with COVID-19 [48], [52].

An HOCl-based antiseptic approach is one of several options for attacking the invading respiratory pathogens upstream of the imminent 2^nd^ infection phase. The current state of knowledge on the efficacy of such antiseptics in the prevention of SARS-CoV-2 infections with various nasal and pharyngeal approaches has been recently summarized [163]. Nasal irrigation with low-level HOCl in patients with common cold demonstrated its antiviral effect against HRV (human rhinovirus) infection of primary human nasal epithelial cells. The results point to the possible operation of innate resistance mechanisms in mucosal epithelia mediated by HOCl, independent of the need for phagocytosis by neutrophils and tissue macrophages [138]. Similar protective contributions by reactive oxygen species (ROS) have been proposed to occur in alveolar cells in the lower respiratory tract [45]. 

HOCl has been shown to exhibit both bactericidal and virucidal activity. For instance, a spray of HOCl solution has demonstrated strong virucidal activity against SARS-CoV-2, with a high safety profile on the nasal and oral mucosa [164], [165].

In addition, HOCl solutions can be applied as hypotonic solutions. Considering that the concentration of electrolytes in the HOCl solution is lower than the average electrolyte concentration of the mucus, electrolytes are absorbed by the mucus (due to the osmotic effect) and reduce its viscosity (softening the nasal mucus despite its counteracting hydrophobic character), thus favoring its clearance and removal [164].

Nasal-spray treatments for respiratory tract viruses have been explored in several pre-clinical and other trials [48], [91], [133], [164], [166], [167], [168]. In these nasal formulations, HOCl has shown bactericidal, fungicidal, or virucidal effects [38], [69], [92], [164], [166], [168], [169], [170], [171], [172]. Several of these antiseptics have demonstrated the ability to diminish the viral load of SARS-CoV-2 by 3–4 lg within 15–30 s *in vitro*. Several such products are already commercially available for prevention or early treatment of COVID-19 and have shown promising results [91], [173], [174]. In this way, HOCl has proven its potential to become a solution for upper respiratory-tract hygiene by assisting intra-cellular defense mechanisms through its extra-cellular attack on adsorbed pathogens (not yet inserted their RNA into intracellular space via endocytosis). 

Reducing the viral load at the ports of entry, i.e., in the nasopharynx, is an important aspect of any epidemic control measure, since the likelihood of attacking the infection increases with the extent of exposure. Since the initial respiratory pathogen load also influences the severity of the disease after infection, biocidal antisepsis during the asymptomatic phase of the infection can even mitigate a manifesting infection during the disease [175]. In this regard, a study by Davies et Al. [176] showed a 4–5 lg reduction of a SARS-CoV-2 titer after treatment with a commercially available mouthwash containing 0.01–0.02 % (100–200 ppm) stabilized HOCl. From a safety perspective, no observable changes in the endoscopic scores were detected after 8 weeks of regular exposure of humans to HOCl via nasal irrigation [166].

A comprehensive review on the antiviral effect of mouthwashes against SARS-COV-2 is presented by Mendoza et al. [177] These studies [167], [178] contribute to the growing body of evidence on the virucidal efficacy of HOCl-containing mouthwashes/oral rinses against SARS-CoV-2, and have important applications in reducing the risk associated with aerosol-generating procedures in dentistry and potentially even for wider infection control.

The oral route of electrolyzed saline in a combined treatment with sinonasal NaOCl irrigation and systemic antibiotics has been reported to overcome a severe skull-base osteitis [178]. 

HOCl as an active virucidal substance for gargling has also been shown to be safe, and has been used to treat viral diarrhea in preclinical trials [179]. Beyond gargling, the addition of HOCl as supplement to drinking water in animal husbandry (e.g., pigs) has shown positive effects as a treatment for infected animals or as a prophylactic against symptom severity in asymptomatic animals [180].

### Inhaling approaches 

Several nebulization and inhaling approaches have been developed to take advantage of the large interaction area of the lungs. As an example, it has been possible to increase mucociliary clearance by inhaling a simple saline solution [181], which also has proven to induce the innate HOCl-based immune response. These inhaling approaches are postulated to have the potential to substantially reduce the pathogen load in the lungs. 

Rasmussen et al. demonstrated that inhalation of an aerosolized HOCl solution by rodents – in accordance with a US-EPA acute 4-hour inhalation toxicity protocol on rats and later an ocular study on rabbits – provided observational, gross pathological, and histopathological evidence that the pulmonary and ocular exposure did not produce different results when compared to control animals [48]. They also demonstrated that even prolonged inhalation of HOCl generates neither acute nor lingering after-effects. Their findings satisfy the standard required by US federal regulatory agencies for determination of the acute toxicity of inhaled drugs or device solutions.

Increasing evidence is emerging of the beneficial effects of inhaling micro-aerosolized hypochlorous acid (HOCl) as a routine intervention in the prevention and treatment of respiratory virus infections, including SARS-CoV-2 [35], [48], [59], [89], [90], [161], [164]. 

The inhalation of nebulized HOCl solutions presents a therapeutic alternative to influence the respiratory epithelium (from the nasopharynx to the lung) during the incubation (viral) phase, by:

- particularly decreasing the viral load at the onset of the disease,
- restraining the progress of the acute (pulmonary) phase, and
- confining the evolution of severe and serious complications.

Decreasing the viral load in the early disease states can change the natural history of the disease.

Two major branches of the prophylactic treatment of respiratory infections are immunoprophylaxis (e.g., vaccines, immunoglobulins) and chemoprophylaxis (e.g., antimicrobial agents). The targets for chemoprophylactic approaches are the inhibition of adhesion, entry, or replication of the pathogen. In the context of respiratory infections, pre- and post-exposure to pathogen prophylactic measures are to be distinguished. HOCl inhalation procedures (a chemoprophylactic approach) demonstrated promising efficacy in both phases [66], [164]. A similar study showed the importance of a significant reduction of viral load in the nasal wash fluids of patients and demonstrated a significant shortening of disease duration and recurrence of symptoms [182].

A recent study with nebulized HOCl-containing solutions and 32 symptomatic subjects showed recovery of blood oxygenation, respiratory rates, and most initial disease-related symptoms. Participants inhaled the solution 4 times a day (15 min each time) for 7 days [59]. The treatments reduced nasal and pharyngeal viral load and can minimize the progression and/or spread of the disease. A series of recent studies on the effect of aerosolized HOCl has demonstrated improved respiration, relief from nasal congestion, relief from sore throat, bronchitis, and a reduction of disease symptoms and severity [48], [133], [164], [168]. 

Delgado-Enciso et al. and Zaizar-Fregoso [133], [134] showed that experimental treatment of COVID-19 with inhaled HOCl decreased the risk of hospitalization by 89% and the risk of death by 96%, and resulted in an 18-fold higher probability of achieving an acceptable symptom status on day 5, compared with conventional medical care alone. Inhaling the HOCl solution markedly reduced the symptomatology and risk of progression in ambulatory patients with COVID-19. Patients received a daily dose of 0.4 mg/d for a total of 10 days. Such a daily dose is about equal to the dose a person would inhale in an atmosphere enriched with 0.02 ppm of HOCl during exposure for 24 hours. This daily exposure is about 10% of the legal limit set by the European Comission [183].

The findings of Delgado-Enciso et al [134] suggest that aerosolized HOCl may protect against aerogenously spread pathogens (e.g., SARS-CoV-2) by reducing the viral load in the upper respiratory tract, thus diminishing the clinical severity of the disease and potential spreading of infection. *In vivo* tests on rabbits confirmed a safe and tolerable profile of the HOCl solution used, documenting its non-irritating characteristics on nasal and oral mucosa. 

Gutiérrez-García et al. [48], [168] showed that naso-pharyngeal and oropharyngeal rinses with neutral electrolyzed water (pH ~7) prevented COVID-19 in non-vaccinated front-line health professionals. The study demonstrated the tremendous effect of aqueous HOCl solutions in protecting against a COVID infection. Daily application (nasal and pharyngeal) of the HOCl solution reduced the disease incidence of non-vaccinated persons by 91%. In the group that received the nasal and oral rinses, the presence of COVID-19-positive cases was 1.2%, while in the group that did not use the rinses (control group), it was 12.7%. The study provided sufficient evidence to postulate that oral or nasopharyngeal application of aqueous HOCl can prevent COVID-19 disease to a large degree. The measured outcome of inhaled, nebulized HOCl indicates that systemic effects result from exposure to HOCl which go beyond a simple antimicrobial effect. 

From an epidemiological point of view, it has been demonstrated that reinfections with the Coronavirus further increase risks of death, hospitalization, and sequelae in multiple organ systems in the acute and post-acute phase [184]. That study also showed that the risks were evident regardless of vaccination status and were most pronounced in the acute phase but also persisted in the post-acute phase at 6 months. This makes a proactive approach to deactivate airborne pathogens, e.g., via vaporized HOCl, even more relevant.

Aerosolized HOCl receives further support from a recent study by Luo et al. [185], who showed that enveloped viruses are prone to inactivation when exposed to strong acidity levels characteristic of atmospheric aerosol. The pH of exhaled infected aerosols decreased by 2–3 points to a pH ~4, which rapidly inactivated the studied influenza-A virus within minutes, whereas SARS-CoV-2 required days. Encouraged by that result, they studied the effect of aerosolization of nitric acid, which caused atmospheric pH to drop by 2 points and decreased inactivation times for both studied viruses by 1 lg. The point is: When indoor air is enriched with acidic HOCl aerosols, the pH will drop and will likely have a similar effect on airborne pathogens, in addition to HOCl’s inherent biocidal effect described above. Luo et al. remarked that “unintentional removal of volatile acids from indoor air by filtration may elevate pH and prolong airborne virus persistence. The overlooked role of aerosol pH has profound implications for virus transmission and mitigation strategies.”

## Regulatory aspects

HOCl is a well-studied, concentration-dependent, non-toxic, non-corrosive, easy to use, and more effective and safer alternative to other chlorine-generating antiseptic agents, such as sodium dichloroisocyanurate (NaDDC) [186]. The concentration limit set by the European Union is 0.22 ppm of active chlorine for constant indoor air exposure indoors [187]. 

HOCl solutions are already included in the WHO list of coronavirus-effective biocides [188] and were entered into the ‘List N’ of United States Environmental Protection Agency (EPA) for use in disinfection against the pandemic coronavirus [189]. 

The European Chemical Agency (ECHA) lists ‘Active Chlorine released from hypochlorous acid (HOCl)’ as a biocidal product applicable in 5 product type areas: Human hygiene, surface disinfection, veterinary hygiene, food, and feed area, and drinking water. HOCl has also been approved by the FDA for:

1. disinfection of food-contact surfaces;
2. high-level disinfection and sterilization of medical instruments;
3. topical applications;
4. use in drinking water; and
5. as a no-rinse food sanitizer [190], [191], [192].

In 2020, due to the need of specific guidance in the context of COVID-19 pandemic, HOCl has also been listed in the following documents as an effective and safe disinfectant or antiseptic:

- The WHO (2020) list of coronavirus-effective biocides (Cleaning and disinfection of environmental surfaces in the context of COVID-19) [188]
- The United States Environmental Protection Agency (EPA) “N” list of Disinfectants for use against SARS-CoV-2 [189]
- The ‘Health Canada’ list of disinfectants with evidence for use against COVID-19 [193]
- The Australian Register of Therapeutic Goods, as a hospital-grade disinfectant effective against COVID-19 [194]
- ECHA – Regulation of allowable HOCl concentration in 5 different product groups [195], [196]

## Conclusions

Historically, carefully controlled HOCl manufacturing methods were costly, which impeded the acceptance and use of HOCl products. However, now that HOCl is available in well-defined, reliable, and economically attractive forms in industrial volumes, the superiority of its performance in disinfection, antisepsis, and wound care argues for its deployment on a much wider scale worldwide [21]. 

HOCl, when used as a sole component within approved limits, shows no negative side effects on living cells in topical, inhaling, and even systemic applications [91]. In terms of the external exposure of individuals to HOCl, it is paramount that such tested, well-documented, and safe threshold levels be respected. 

The molecular and biochemical pathways of HOCl are complex, whether they involve direct interaction with epithelial tissues or mediate and trigger certain steps of infection cascades. The reviewed studies show no negative tissue response or major adverse effects. HOCl inactivates viruses, bacteria, endospores, and fungi, is safe for human tissues (including eye, lung, and skin), environmentally benign and thus requires no toxic waste disposal or hazardous material management, and yet can degrade the infectivity even of prions and virions at high log-reduction values (LVR) of >5 [2].

Many studies clearly show its high potential for peri-operative antisepsis, wound care, and more recently, for nasopharyngeal sprays and inhalation procedures. Wound-care procedures using HOCl are already recommended medical practice. The use of HOCl to both prevent and contain infectious diseases transmitted mainly via air-borne pathogens has seen a remarkable increase of attention and research focus. Many studies show the high potential of nasopharyngeal sprays and inhalation procedures to aid infection control. But the use in humans is not undisputed. Many organizations categorically distance themselves from its use as preventive or therapeutic antiseptic for external or internal application; some even recommend its categorical ban [197], [198]. There are numerous studies on the negative and potentially harmful effects of chlorination stress. The safety of any HOCl application is of course the utmost concern. 

The Corona pandemic has provided the main motivation for intensified research activities on the potential use of HOCl to fight airborne pathogens, which otherwise would not have been adequately recognized. The overall safety profile of HOCl, recognition of its *in vitro* efficacy, and popular understanding of HOCl as a natural product of human cellular defense, have each encouraged new use patterns. Further expansion of HOCl as an environmental disinfectant is both appropriate and inevitable, and studies are beginning to provide increasing evidence about the beneficial results [48]. 

The potential significance of the use of HOCl as a therapeutic and possibly even prophylactic tool in the management of COVID-19 patients (and beyond this disease) is substantial. Administration of aerosolized HOCl could offer an important advance in patient care. It is also worth noting that the specific amino-acid residues in the SARS-CoV-2 spike protein, against which the redox mechanism of action for HOCl is active, are conserved in all variants evaluated to date. Each variant identified so far is likely to be similarly susceptible, and related research is underway. Due to increasing morbidity and mortality seen with variant SARS-CoV-2 strains, along with the availability of HOCl formulations suitable for systemic administration in humans, particularly through inhalation, more extensive clinical evaluation should receive priority [48]. 

The potential of HOCl to serve as a safe, efficient, and cost-effective indoor disinfectant for use in prevention and symptom containment leads us far beyond COVID related applications.

Futher information on *HOCl in indoor atmospheres*, see Attachment 1.

## Notes

### Competing interests

The authors are affiliated with oji Europe GmbH, a company which develops air- and surface disinfection systems.

In particular:

- Dr. Dr. Dirk Boecker is head of oji’s scientific advisory committee
- Mr. Zhentian Zhang is a consultant to the company
- Dr. Roland Breves is head of the HENKEL microbiology lab where oji conducts research experiments and is a member of oji’s scientific advisory panel
- Prof. Dr. Felix Herth is head of the Thorax Clinic in Heidelberg and acts as a medical advisor to oji Europe
- Prof. Dr. Clemens Bulitta is a member of oji’s scientific advisory panel

Prof. Dr. Axel Kramer is not affiliated with oji nor has received any remuneration or benefits from the company. 

### ORCID

These are the authors’ ORCID IDs:

- Dirk Boecker: 0000-0002-4271-5595
- Zhenitan Zhang: 0000-0001-7781-6313
- Roland Breves: 0009-0003-9963-765X
- Felix Herth: 0000-0002-7638-2506
- Axel Kramer: 0000-0003-4193-2149
- Clemens Bulitta: 0009-0000-4405-4578

## Supplementary Material

HOCl in indoor atmospheres

## Figures and Tables

**Table 1:**
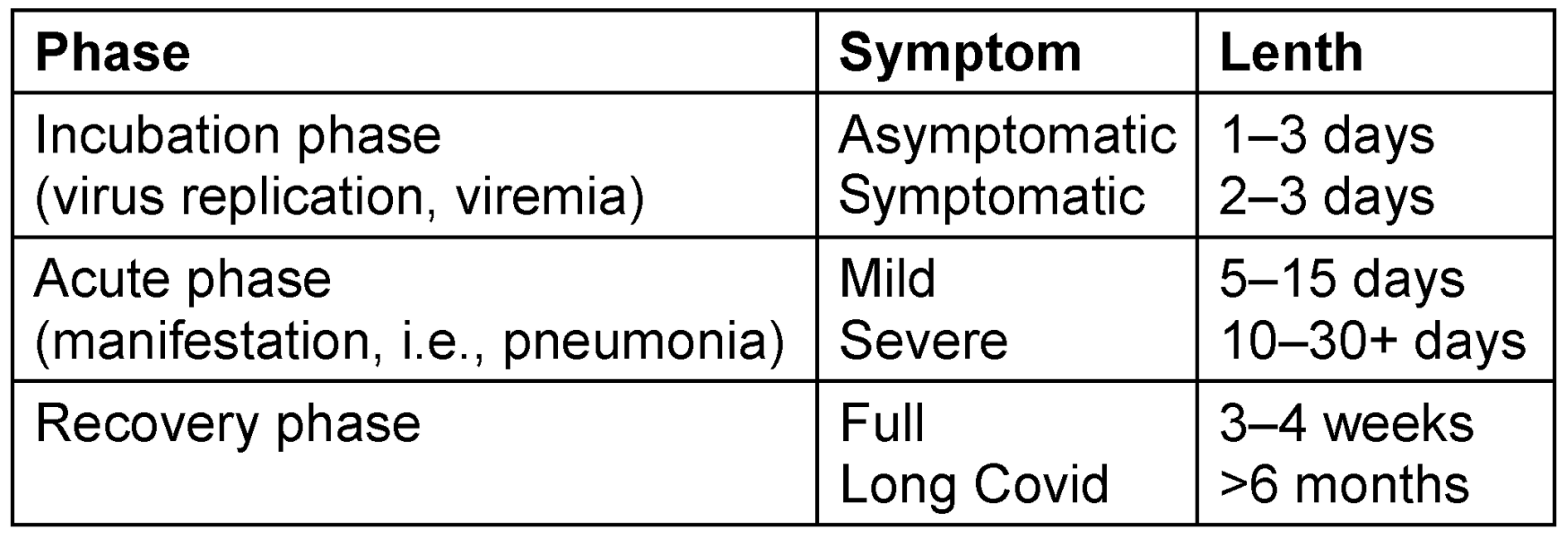
Phases and lengths of disease states of COVID-19

**Table 2:**
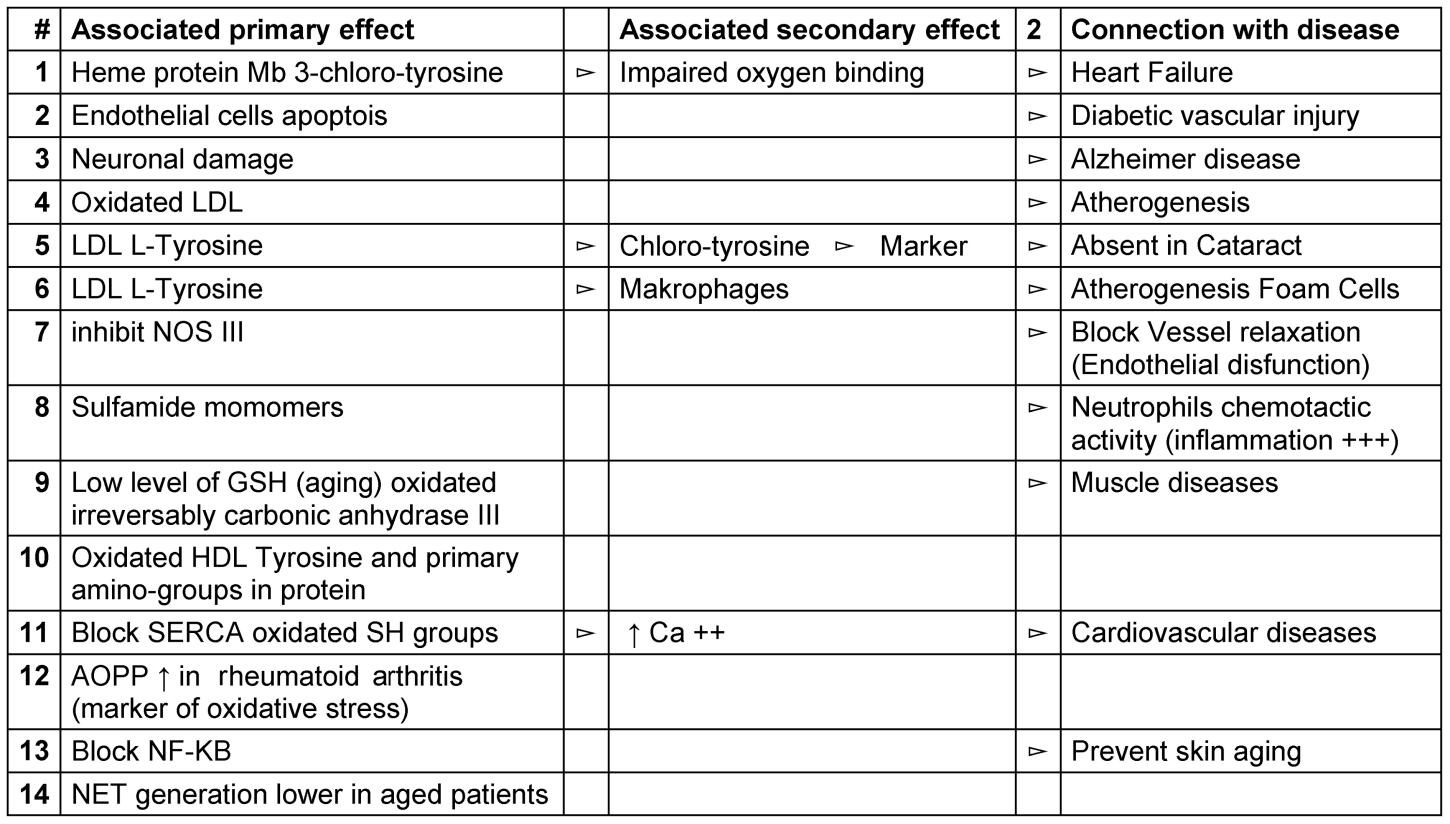
Overview of age-related diseases and underlying pathological reactions caused by an exceedingly high and out-of-control endogenous HOCl concentration (see Casciaro et al. [108])
