# Supplementary material for: Antimicrobial efficacy, mode of action and in vivo use of hypochlorous acid (HOCl) for prevention or therapeutic support of infections
Source: GMS Hyg Infect Control. 2023 Mar 27;18:Doc07. doi: 10.3205/dgkh000433 (PMC10073986; doi:10.3205/dgkh000433)
Supplement: HOCl in indoor atmospheres [file HIC-18-07-s-001.pdf]

# Appendix – HOCl in indoor atmospheres

The focus is laid on the creation of radicals and downstream reaction cascades driven by chlorine [Cl•] and hydroxyl [HO•] radicals created by the decay of HOCl. The oxidative power of gaseous HOCl must be understood as the combined action of HOCl itself and its two initial 'descendants', the hydroxyl [HO•] and chlorine [Cl•] radicals.

**Background:** Indoor air quality is influenced by the contents and activities within a building, along with the properties of the building itself and its environmental setting. Good air quality is typically characterized by the lack of pollutants like airborne particles and harmful trace gases. The concentration of any component of indoor air is set by a balance between the rates of production and removal. Primary sources of trace gases and particulate matter (PM) include cooking, cleaning, building materials, and commercial products.

Secondary chemistry can transform primary pollutants, especially when oxidant levels are elevated. Whereas much of outdoor atmospheric chemistry is driven by photo-oxidation processes, indoor environments may be subject to lower light (except near windows in direct sunlight) and a high surface area-to-volume ratio. Thus, the development of organic and aqueous films on indoor surfaces strongly influences indoor air as they mediate multiphase chemical reactions and equilibrium partitioning of semi-volatile components." [117]

Indoor gas-phase chemistry occurs with ozone ($O_3$), hydroxyl (HO•) radicals, and other oxidants. $O_3$ is mainly introduced into the indoor environment from outdoor air or commercial products, while HO• radicals can be chemically produced indoors. Secondary oxidation chemistry removes some compounds (e.g., alkenes), but produces others (e.g., aldehydes, peroxides), and can lead to an increased mass concentration of particulate matter and increased number concentration of ultrafine aerosol. While human breath is a source of biological particulate matter (PM) of relevance for transmission of some diseases, human lungs are also efficient sinks of particles [117]

Vaporized HOCl is a strong oxidizing agent and reacts (deactivates) with many types of pathogens, e.g.: the reaction with coronavirus is happening at the outer layer of the virus, particularly the spike proteins, which are modified and become non-reactive.

**HOCl and its oxidation cascade:** Reaction and decay of HOCl: Airborne HOCl decays with rates of 0.3–4%/min [17–91%/h] depending on a couple of factors like surface characteristics, sunlight, temperature, oxygen concentration, humidity, air exchange rates, and others. The main decay route runs along formation of Cl• radicals, which in turn react with volatile or surface laden organic material:

$$HOCl + h\nu \rightarrow Cl\bullet + OH\bullet$$

Chlorine radicals produced photolytically may react with and oxidize volatile organic compounds (VOC = RH) and leave a reduced chloride anion to form HCl:

$$Cl\bullet + RH \rightarrow R\bullet + HCl$$

The hydroxyl radical can react with HCl to resubstitute a Cl radical

$$OH\bullet + HCl \rightarrow Cl\bullet + H_2O$$

Unlike OH•, chlorine radicals are not regenerated within the oxidation cycle. However, chlorine radicals can be regenerated by heterogeneous cycling from chloride containing aerosols. The net effect is:

$$HOCl + 2RH \rightarrow 2R\bullet + HCl + H_2O$$

Attachment to: Boecker D, Zhang Z, Breves R, Herth F, Kramer A, Bulitta C. Antimicrobial efficacy, mode of action and in vivo use of hypochlorous acid (HOCl) for prevention or therapeutic support of infections. GMS Hyg Infect Control. 2023;18:Doc07.
DOI: 10.3205/dgkh000433

This also reflects the high oxidative potential of airborne HOCl molecules towards volatile organic compounds. In essence HOCl oxidizes airborne or surface laden organic material (e.g.: bacteria, viruses, other pathogens) forming R• components, which in turn react towards higher and usually less reactive oxidized forms. The progressive breakdown of VOC and the oxidation by-products ultimately leads to a reduction in total VOC, thereby improving air quality.

The HOCl reach in indoor environments is very substantial, even at high decay rates of 3%/min (Figure 4).

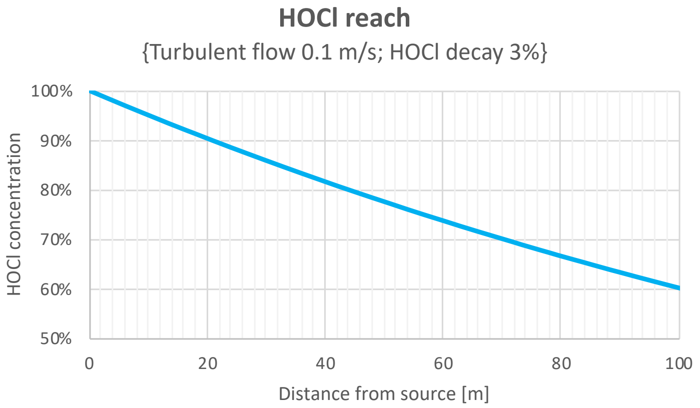


**Figure 4: 1st order estimate of room concentration gradient in case of a turbulent flow pattern**

Independent studies on the effect of the hydroxyl radical – which is part of the HOCl reaction chain – conducted by Columbia Laboratories demonstrated that the total VOC levels were reduced, and steady state concentrations of oxidation by-products remained within the low ppb range, at or near ambient levels, and did not accumulate[199].

The deactivation of gaseous HOCl in environments which are free of organic components (VOCs and surface laden contaminants) is reduced to basically three processes:

- Photochemical transformation to Cl• radicals
- Catalytic degradation on metal carrying indoor surfaces
- Reaction with water vapor to dissociate into $H^+$– and $OCl^-$ – ions

**Creation of Hydroxyl (HO•) and Chlorine (Cl•) radicals:** The hydroxyl cascade is like the HOCl induced reaction cascade [200], [201]. The difference is that the HOCl molecules are way more stable (they ‘decay’ [= form radicals]) with 2–3% /min) and therefore reach ‘far’ points in rooms themselves.

$$HOCl + h\nu \rightarrow Cl\bullet + HO\bullet$$

Chlorine radicals produced photolytically may react with and oxidize volatile organic compounds (VOC = RH) and leave a reduced Chlor anion to form HCl:

$$Cl\bullet + RH \rightarrow R\bullet + HCl$$

The hydroxyl radical can react with HCl to resubstitute a Cl radical

$$HO\bullet + HCl \rightarrow Cl\bullet + H_2O$$

Hydroxyl and chlorine radicals are extremely reactive (high redox potential) and thus extremely short lived and don’t accumulate. The indoor reactivity is only based on their ability to initiate a cascade of redox reactions where one radical transmits its redox power in a chain reaction where involved radicals have an ever-decreasing redox potential, but an increasing lifetime. In this way the redox power of the initial hydroxyl and chlorine radicals can ‘travel’ relatively large indoor distances.

Attachment to: Boecker D, Zhang Z, Breves R, Herth F, Kramer A, Bulitta C. Antimicrobial efficacy, mode of action and in vivo use of hypochlorous acid (HOCl) for prevention or therapeutic support of infections. GMS Hyg Infect Control. 2023;18:Doc07. DOI: 10.3205/dgkh000433

It is important to note that the hydroxyl radical itself belongs to a group of reactive oxygen species (ROS), which in biological systems can cause primary molecular damage, particularly concerning proteins or membrane lipids – because of a lack of specific counter-acting system. When created in indoor atmospheres, it almost immediately transfers its oxidative potential onto air-borne organic components with retain a reduced redox potential and so allow to mediate and safeguard the hydroxyl's original strength [202].

The resulting 'cascade redox power' can disinfect airborne VOCs as well as surface bound pathogens. Unlike the situation with HOCl, the hydroxyl effect cannot be measured by indoor sensors. The necessary amount must be estimated based on lab experiments.

**Hydroxyl radicals:** There are, on average, 3 to 10 million hydroxyl radicals (HO•) in each cubic centimeter of ambient outdoor air during sunny daylight hours [203], [204], [205]. Even at these relatively low concentrations, hydroxyls are the primary environmental purifying agent [206]. Hydroxyls neutralize most natural and man-made pollutants including isoprene, methane, benzene, toluene, aromatics, hydrogen sulfide, ammonia, and ozone [206], [207]. Hydroxyls decompose volatile organic compounds (VOC) to produce carbon dioxide and water [208]. Indoor environments do not naturally contain hydroxyl radicals as hydroxyls generated outdoors react too rapidly with volatile chemicals to diffuse indoors.

Hydroxyl is created in the atmosphere along the following processes [209]:

$$H_20 + h\nu \rightarrow HO\bullet + H\bullet$$

$$H\bullet + H_20 \rightarrow HO\bullet + H_2 \text{ or}$$

$$H\bullet + O_2 \rightarrow HO\bullet + O\bullet$$

$$O\bullet + H_2O \rightarrow 2\ HO\bullet$$

The decomposition of volatile organic and inorganic compounds by hydroxyl radicals involves a complex series of free radical oxidation steps that gradually result in the loss of individual carbon atoms to eventually form carbon dioxide ($CO_2$).

Hydroxyl ions are extremely short-lived molecules. They usually exist for no longer than a second or so before becoming involved in an air-cleansing chemical reaction. The process is very complex but continues to completion because the reactions involved are highly favored thermodynamically and kinetically. The rates of reactions of hydroxyls with VOCs are very fast. That is precisely the reason why it has been so difficult to accurately measure hydroxyl levels in the earth's atmosphere.

Hydroxyl molecules don't accumulate indoors. They rapidly react with microorganisms and nearly all volatile organic chemicals that exist in air.

Given the speed of reaction, most hydroxyls react inside or near the exhaust of a hydroxyl generating device (e.g.: an instrument from the company PYURE®), while the organic peroxy- and oxy-radicals are more stable than hydroxyls and have time to diffuse out of the device and circulate throughout a treated space.

The hydroxyl radical is one of the most powerful oxidants found in nature, surpassed only by atomic fluorine in its oxidation potential. As an efficient oxidant, hydroxyls are driven to regain one electron to restore their very stable valence configuration. The most facile way to accomplish this in an environment rich in VOCs is to abstract a hydrogen atom with its single electron, leaving behind an organic free radical. The newly formed organic free radical has an unpaired electron that is initially localized on the carbon atom:

$$R\text{-}CH_2\text{-}CH_2\cdot$$

This moiety rapidly reacts with ambient oxygen to form a peroxy free radical in which the free electron is now localized on an oxygen atom:

$$R\text{-}CH_2\text{-}CH_2\text{-}O\text{-}O\cdot$$

Attachment to: Boecker D, Zhang Z, Breves R, Herth F, Kramer A, Bulitta C. Antimicrobial efficacy, mode of action and in vivo use of hypochlorous acid (HOCl) for prevention or therapeutic support of infections. GMS Hyg Infect Control. 2023;18:Doc07. DOI: 10.3205/dgkh000433

This species is a powerful oxidant and will react with VOCs and inorganic gases. It is more stable than the hydroxyl radical and able to travel further away from the reaction chamber to react with VOCs and microorganisms in the treatment area. Peroxy radicals can also rapidly form oxy radicals in ambient air:

$$R\text{-}CH_2\text{-}CH_2\text{-}O\cdot$$

Oxy radicals are powerful oxidants and even more stable than their per-oxy counterparts, and therefore can travel even further throughout the treatment space to react with microorganisms and other VOCs.

These types of intermediate oxidants are how ODOROX® systems can penetrate and treat structures and contents at a distance from the device.

A kind of cascade effect takes hold: An ongoing free radical chain reaction sequence and the formation of a series of intermediate oxidants. The free radical transfer reactions essentially 'unzip' carbon chains rapidly. If hydroxyls are continuously formed, both thermodynamic and kinetic factors favor continued oxidation of VOCs. In the cascade effect, successive generations of oxidants continue to decompose and dissociate VOC carbon chains creating $CO_2$ along the 'deconstruction' way.

Studies of the Lovelace Respiratory Research Institute (LRRI) and Columbia Labs, along with a very significant number of field experiences, have confirmed that the hydroxyl radicals and its 'descendant R• radicals' safely and effectively reduce ambient odor and VOC concentrations indoors [199]. In a study of Aerosol Laboratories (Olathe, KS, USA) it has been demonstrated that hydroxyl-initiated R• radical cascade disinfection effectively decontaminated surfaces by 99.99%–99.999% (log factors 4–5). The study included two vegetative bacteria, two viruses, a fungal spore, and a bacterial endospore as simulants for a broader range of pathogenic organisms [210].

**Ozone aspects:** The production of hydroxyl radicals also creates ozone. This ozone progressively decomposes in water bearing atmospheres partly into hydroxyl radicals, which themselves form complex intermediate functional agents with $O_3$ [211]:

$$m\ O_3 + n\ H_2O \rightarrow \ldots \rightarrow \sim m/2\ OH\bullet + O_3/OH\bullet$$

**Formation of HCl:** Chlorine gas can disproportionate into hypochlorous acid and hydrogen chloride [212]:

$$Cl_2 + H_2O \leftrightarrow HOCl + HCl$$

In this reaction the chlorine is disproportionated into its –1 and +1 oxidation state. HOCl becomes a very active oxidation substance. The second chlorine loses all further oxidative (ala disinfecting) capability. The formed HCl is ultimately dissolved in air- or surface-humidity:

$$HCl + H_2O \leftrightarrow H_3O+ + Cl^-$$

Attachment to: Boecker D, Zhang Z, Breves R, Herth F, Kramer A, Bulitta C. Antimicrobial efficacy, mode of action and in vivo use of hypochlorous acid (HOCl) for prevention or therapeutic support of infections. GMS Hyg Infect Control. 2023;18:Doc07. DOI: 10.3205/dgkh000433

## References

1. Pratt HT. Role of Javelle water in the history of chlorine bleaching. Bibliographic database of the Conservation Information Network (BCIN). 1989 [last access 2023 Feb 10]. Available from: https://www.bcin.ca/bcin/detail.app?id=105890&

2. Williams J, Rasmussen E, Robins L. Hypochlorous Acid: Harnessing an Innate Response. The Infection Prevention Strategy (TIPS). 2017;1-9. Available from: https://www.infectioncontrol.tips/2017/10/06/hypochlorous-innate-response/?format=pdf

3. Fuchs B. Hypochlorsäure in vivo und in vitro: Rolle im Immunsystem und als Desinfektionsmittel. Abschlussarbeit Postgradualstudium Toxikologie Universität Leipzig. Leipzig: Universität Leipzig; 2006.

4. Edward DG, Lidwell OM. Studies on air-borne virus infections: III. The killing of aerial suspensions of influenza virus by hypochlorous acid. J Hyg (Lond). 1943 Sep;43(3):196-200. DOI: 10.1017/s002217240001281x

5. Mackie TJ. The problem of air-borne infection. Edinb Med J. 1942;49(10):607-27.

6. Masterman AT. Air purification by hypochlorous acid gas. J Hyg (Lond). 1941 Jan;41(1):44-54.1. DOI: 10.1017/s0022172400012286

7. Elford WJ, van den Ende J. Studies on the disinfecting action of hypochlorous acid gas and sprayed solution of hypochlorite against bacterial aerosols. J Hyg (Lond). 1945 Jan;44(1):1-14. DOI: 10.1017/s0022172400013255

8. Wells WF. Air-borne infection and sanitary air control. J Ind Hyg. 1935; 17:253-7.

9. Twort CC, Baker AH, Finn SR, Powell EO. The disinfection of closed atmospheres with germicidal aerosols. J Hyg (Lond). 1940 May;40(3):253-344.5. DOI: 10.1017/s0022172400027820

10. Pulvertaft RJ, Walker JW. The control of air-borne bacteria and fungus spores by means of aerosols. J Hyg (Lond). 1939 Nov;39(6):696-704. DOI: 10.1017/s0022172400059635

11. Pulvertaft RJ v, Walker JW. The control of air-borne bacteria and fungus spores by means of aerosols. Lancet. 1939;233(6026):443-6. doi: 10.1016/S0140-6736(00)77618-5

12. Masterman AT. Air purification in inhabited rooms by spraying or atomizing hypochlorites. J Ind Hyg Toxicol. 1938;20:278-88.

13. Scheffler S. Report: Human and dietary risk assessment for hypochlorous acid, nebulized by an atomizer. Hannover: Fraunhofer Institute of Toxicology and Experimental Medicine; 2020 [last access 2023 Feb 10]. Available from: https://www.bartelt.at/wp-content/uploads/2021/01/Fraunhofer-human-and-dietary-risk-assessment-Hypochlorsaeure-Eng.pdf

14. ECHA. Product-types - ECHA. 2020 [last access 2023 Feb 10]. Available from: https://echa.europa.eu/regulations/biocidal-products-regulation/product-types

15. Lu MC, Chen PL, Huang DJ, Liang CK, Hsu CS, Liu WT. Disinfection efficiency of hospital infectious disease wards with chlorine dioxide and hypochlorous acid. Aerobiologia (Bologna). 2021;37(1):29-38. DOI: 10.1007/s10453-020-09670-8

16. Hughson AG, Race B, Kraus A, Sangaré LR, Robins L, Groveman BR, Saijo E, Phillips K, Contreras L, Dhaliwal V, Manca M, Zanusso G, Terry D, Williams JF, Caughey B. Inactivation of Prions and Amyloid Seeds with Hypochlorous Acid. PLoS Pathog. 2016 Sep;12(9):e1005914. DOI: 10.1371/journal.ppat.1005914

17. Remucal CK, Manley D. Emerging investigators series: the efficacy of chlorine photolysis as an advanced oxidation process for drinking water treatment. Environ Sci (Camb). 2016;2(4):565-79. DOI: 10.1039/C6EW00029K

18. Takeda Y, Uchiumi H, Matsuda S, Ogawa H. Acidic electrolyzed water potently inactivates SARS-CoV-2 depending on the amount of free available chlorine contacting with the virus. Biochem Biophys Res Commun. 2020 Sep;530(1):1-3. DOI: 10.1016/j.bbrc.2020.07.029

19. Snell JA, Jandova J, Wondrak GT. Hypochlorous Acid: From Innate Immune Factor and Environmental Toxicant to Chemopreventive Agent Targeting Solar UV-Induced Skin Cancer. Front Oncol. 2022;12:887220. DOI: 10.3389/fonc.2022.887220

20. USDA. Hypochlorous Acid. Technical Evaluation Report. Washington, D.C.: USDA; 2015. p. 1-24. Available from: https://www.ams.usda.gov/sites/default/files/media/Hypochlorous%20Acid%20TR%2008%2013%2015.pdf

21. Rasmussen E. Hypochlorous acid (HOCl) for disinfection, antisepsis, and wound care in core categories 15.1, 15.2, and 13. Application for Inclusion in the 2021 WHO Essential Medicines List in Core Categories. 2020. p. 1-57. Available from: https://cdn.who.int/media/docs/default-source/essential-medicines/2021-eml-expert-committee/applications-for-addition-of-new-medicines/a.18_hypochlorous-acid.pdf?sfvrsn=35222172_4

22. Robins LI, Clark A, Gafken PR, Alam S, Milici J, Hassan R, Wang CY, Williams J, Meyers C. Hypochlorous acid as a disinfectant for high-risk HPV: Insight into the mechanism of action. J Med Virol. 2022 Jul;94(7):3386-93. DOI: 10.1002/jmv.27716

23. Meyers J, Ryndock E, Conway MJ, Meyers C, Robison R. Susceptibility of high-risk human papillomavirus type 16 to clinical disinfectants. J Antimicrob Chemother. 2014 Jun;69(6):1546-50. DOI: 10.1093/jac/dku006

24. Severing AL, Rembe JD, Koester V, Stuermer EK. Safety and efficacy profiles of different commercial sodium hypochlorite/hypochlorous acid solutions (NaClO/HClO): antimicrobial efficacy, cytotoxic impact and physicochemical parameters in vitro. J Antimicrob Chemother. 2019 Feb;74(2):365-372. DOI: 10.1093/jac/dky432

25. BAG. Anerkannte Aufbereitungsverfahren für Trinkwasser. Bern: Bundesamt für Gesundheit; 2010. p. 107. Available from: https://www.blv.admin.ch/dam/blv/de/dokumente/lebensmittel-und-ernaehrung/lebensmittelsicherheit/verantwortlichkeiten/anerkannte-aufbereitungsverfahren-trinkwasser.pdf.download.pdf/anerkannte-aufbereitungsverfahren-trinkwasser.pdf

26. Butterfield CT, Wattie E, Megregian S, Chambers CW. Influence of pH and temperature on the survival of coliforms and enteric pathogens when exposed to free chlorine. Public Health Rep. 1943;58(51):1837. DOI: 10.2307/4584715

27. HOCl HAWAII. What is hypochlorous acid? FAQs. 2022 [last access 2023 Feb 10]. Available from: https://hoclhawaii.com/pages/faqs

28. Fidler A, Steyer A, Manevski D, Gašperšič R. Virus transmission by ultrasonic scaler and its prevention by antiviral agent: An in vitro study. J Periodontol. 2022 Jul;93(7):e116-e124. DOI: 10.1002/JPER.21-0335

29. Murashevych B, Girenko D, Maslak H, Stepanskyi D, Abraimova O, Netronina O, Zhminko P. Acute inhalation toxicity of aerosolized electrochemically generated solution of sodium hypochlorite. Inhal Toxicol. 2022;34(1-2):1-13. DOI: 10.1080/08958378.2021.2013348

30. Miyagawa M. [Globally harmonized system of classification and labelling of chemicals (GHS) and its implementation in Japan]. Nihon Eiseigaku Zasshi. 2010 Jan;65(1):5-13. DOI: 10.1265/jjh.65.5

31. Squadrito GL, Postlethwait EM, Matalon S. Elucidating mechanisms of chlorine toxicity: reaction kinetics, thermodynamics, and physiological implications. Am J Physiol Lung Cell Mol Physiol. 2010 Sep;299(3):L289-300. DOI: 10.1152/ajplung.00077.2010

32. Jandova J, Snell J, Hua A, Dickinson S, Fimbres J, Wondrak GT. Topical hypochlorous acid (HOCl) blocks inflammatory gene expression and tumorigenic progression in UV-exposed SKH-1 high risk mouse skin. Redox Biol. 2021 Sep;45:102042. DOI: 10.1016/j.redox.2021.102042

33. Ulfig A, Leichert LI. The effects of neutrophil-generated hypochlorous acid and other hypohalous acids on host and pathogens. Cell Mol Life Sci. 2021 Jan;78(2):385-414. DOI: 10.1007/s00018-020-03591-y

34. Lapenna D, Cuccurullo F. Hypochlorous acid and its pharmacological antagonism: an update picture. Gen Pharmacol. 1996 Oct;27(7):1145-7. DOI: 10.1016/s0306-3623(96)00063-8

35. Wang L, Bassiri M, Najafi R, Najafi K, Yang J, Khosrovi B, et al. Hypochlorous acid as a potential wound care agent part i. stabilized hypochlorous acid: a component of the inorganic armamentarium of innate immunity. J Burns Wounds. 2007;6(e5):65-79.

36. Ramalingam S, Cai B, Wong J, Twomey M, Chen R, Fu RM, Boote T, McCaughan H, Griffiths SJ, Haas JG. Antiviral innate immune response in non-myeloid cells is augmented by chloride ions via an increase in intracellular hypochlorous acid levels. Sci Rep. 2018 Sep;8(1):13630. DOI: 10.1038/s41598-018-31936-y

37. Cohen JL, Gold MH. Integrating HOCl into wound care: a new paradigm for patient management. Clin insights. 2017;(7):1-2.

38. Bale B, Doneen AL, Hight T, Vigerust DJ. Enhancement of innate immunity to COVID-19 with natural measures. Immun Res. 2020;16(3):1-4. DOI: 10.35248/17457580.19.1673

39. Ashby MT, Carlson AC, Scott MJ. Redox buffering of hypochlorous acid by thiocyanate in physiologic fluids. J Am Chem Soc. 2004 Dec;126(49):15976-7. DOI: 10.1021/ja0438361

40. Ashby MT. Chapter 8 – Hypothiocyanite. In: van Eldik R, Ivanović-Burmazović I, editors. Advances in Inorganic Chemistry: Inorganic/Bioinorganic Reaction Mechanisms. London: Academic Press; 2012. p. 263-303. DOI: 10.1016/B978-0-12-396462-5.00008-8

41. Kalmár J, Lente G, Fábián I. Detailed kinetics and mechanism of the oxidation of thiocyanate ion (SCN-) by peroxomonosulfate ion (HSO5(-)). Formation and subsequent oxidation of hypothiocyanite ion (OSCN-). Inorg Chem. 2013 Feb;52(4):2150-6. DOI: 10.1021/ic302544y

42. Lorenzen I, Mullen L, Bekeschus S, Hanschmann EM. Redox Regulation of Inflammatory Processes Is Enzymatically Controlled. Oxid Med Cell Longev. 2017;2017:8459402. DOI: 10.1155/2017/8459402

43. Sakarya S, Gunay N, Karakulak M, Ozturk B, Ertugrul B. Hypochlorous acid: An ideal wound care agent with powerful microbicidal, antibiofilm, and wound healing potency. Wounds. 2014;26(12):342-50.

44. Nguyen K, Bui D, Hashemi M, Hocking DM, Mendis P, Strugnell RA, Dharmage SC. The Potential Use of Hypochlorous Acid and a Smart Prefabricated Sanitising Chamber to Reduce Occupation-Related COVID-19 Exposure. Risk Manag Healthc Policy. 2021;14:247-52. DOI: 10.2147/RMHP.S284897

45. Evans SE, Xu Y, Tuvim MJ, Dickey BF. Inducible innate resistance of lung epithelium to infection. Annu Rev Physiol. 2010;72:413-35. DOI: 10.1146/annurev-physiol-021909-135909

46. Zgliczyński JM, Stelmaszyńska T, Domański J, Ostrowski W. Chloramines as intermediates of oxidation reaction of amino acids by myeloperoxidase. Biochim Biophys Acta (Enzymology. 1971;235(3):419-24. DOI: 10.1016/0005-2744(71)90281-6

47. Huxtable RJ. Physiological actions of taurine. Physiol Rev. 1992 Jan;72(1):101-63.

DOI: 10.1152/physrev.1992.72.1.101

48. Rasmussen E, Robins L, Williams J. Inhalation of a fog of hypochlorous acid (HOCl): Biochemical, antimicrobial, and pathological assessment. Arch Intern Med Res. 2022;5(3):311-8. DOI: 10.26502/aimr.0111

49. Fukuyama T, Martel BC, Linder KE, Ehling S, Ganchingco JR, Bäumer W. Hypochlorous acid is antipruritic and anti-inflammatory in a mouse model of atopic dermatitis. Clin Exp Allergy. 2018 Jan;48(1):78-88. DOI: 10.1111/cea.13045

50. Gottardi W, Nagl M. Chemical properties of N-chlorotaurine sodium, a key compound in the human defence system. Arch Pharm (Weinheim). 2002 Nov;335(9):411-21. DOI: 10.1002/1521-4184(200212)335:9<411::AID-ARDP411>3.0.CO;2-D

51. Lackner M, Rössler A, Volland A, Stadtmüller MN, Müllauer B, Banki Z, Ströhle J, Luttick A, Fenner J, Sarg B, Kremser L, Tone P, Stoiber H, von Laer D, Wolff T, Schwarz C, Nagl M. N-chlorotaurine is highly active against respiratory viruses including SARS-CoV-2 (COVID-19). Emerg Microbes Infect. 2022 Dec;11(1):1293-1307. DOI: 10.1080/22221751.2022.2065932

52. de la Rica R, Borges M, Gonzalez-Freire M. COVID-19: In the Eye of the Cytokine Storm. Front Immunol. 2020;11:558898. DOI: 10.3389/fimmu.2020.558898

53. Rasmussen KH, Hawkins CL. Role of macrophage extracellular traps in innate immunity and inflammatory disease. Biochem Soc Trans. 2022 Feb;50(1):21-32. DOI: 10.1042/BST20210962

54. Robins LI, Keim EK, Robins DB, Edgar JS, Meschke JS, Gafken PR, Williams JF. Modifications of IL-6 by Hypochlorous Acids: Effects on Receptor Binding. ACS Omega. 2021 Dec;6(51):35593-9. DOI: 10.1021/acsomega.1c05297

55. van Doremalen N, Bushmaker T, Morris DH, Holbrook MG, Gamble A, Williamson BN, Tamin A, Harcourt JL, Thornburg NJ, Gerber SI, Lloyd-Smith JO, de Wit E, Munster VJ. Aerosol and Surface Stability of SARS-CoV-2 as Compared with SARS-CoV-1. N Engl J Med. 2020 Apr;382(16):1564-7. DOI: 10.1056/NEJMc2004973

56. Galvin S, Boyle M, Russell RJ, Coleman DC, Creamer E, O'Gara JP, Fitzgerald-Hughes D, Humphreys H. Evaluation of vaporized hydrogen peroxide, Citrox and pH neutral Ecasol for decontamination of an enclosed area: a pilot study. J Hosp Infect. 2012 Jan;80(1):67-70. DOI: 10.1016/j.jhin.2011.10.013

57. Marr L, Miller S, Prather K, Haas C, Bahnfleth W, Corsi R, Tang J, Herrmann H, Politt K, Ballester J, Jimenez JL. FAQs on Protecting Yourself from Aerosol Transmission. Version: 1.88. 2021 13 Aug [last access 2023 Feb 10]. Available from: https://tinyurl.com/FAQ-aerosols

58. Greenhalgh T, Jimenez JL, Prather KA, Tufekci Z, Fisman D, Schooley R. Ten scientific reasons in support of airborne transmission of SARS-CoV-2. Lancet. 2021 May;397(10285):1603-5. DOI: 10.1016/S0140-6736(21)00869-2

59. González RCR, López ET. Super-oxidized solution nebulization for the symptomatic treatment of airway infections including COVID-19. Cases Rep. 2020;5(8):299-308. DOI: 10.23958/ijirms/vol05-i08/922

60. Weers J. Inhaled antimicrobial therapy – barriers to effective treatment. Adv Drug Deliv Rev. 2015 May;85:24-43. DOI: 10.1016/j.addr.2014.08.013

61. Elphick M, von Hollen D, Pritchard JN, Nikander K, Hardaker LE, Hatley RH. Factors to consider when selecting a nebulizer for a new inhaled drug product development program. Expert Opin Drug Deliv. 2015 Aug;12(8):1375-87. DOI: 10.1517/17425247.2015.1014339

62. Safdar A, Shelburne SA, Evans SE, Dickey BF. Inhaled therapeutics for prevention and treatment of pneumonia. Expert Opin Drug Saf. 2009 Jul;8(4):435-49. DOI: 10.1517/14740330903036083

63. RKI. Epidemiologischer Steckbrief zu SARS-CoV-2 und COVID-19. Berlin: RKI; 2021

Jan 26 [last access 2023 Feb 10]. Available from: https://www.rki.de/DE/Content/InfAZ/N/Neuartiges_Coronavirus/Steckbrief.html

64. To KK, Tsang OT, Leung WS, Tam AR, Wu TC, Lung DC, Yip CC, Cai JP, Chan JM, Chik TS, Lau DP, Choi CY, Chen LL, Chan WM, Chan KH, Ip JD, Ng AC, Poon RW, Luo CT, Cheng VC, Chan JF, Hung IF, Chen Z, Chen H, Yuen KY. Temporal profiles of viral load in posterior oropharyngeal saliva samples and serum antibody responses during infection by SARS-CoV-2: an observational cohort study. Lancet Infect Dis. 2020 May;20(5):565-74. DOI: 10.1016/S1473-3099(20)30196-1

65. Liu L, Wei Q, Alvarez X, Wang H, Du Y, Zhu H, Jiang H, Zhou J, Lam P, Zhang L, Lackner A, Qin C, Chen Z. Epithelial cells lining salivary gland ducts are early target cells of severe acute respiratory syndrome coronavirus infection in the upper respiratory tracts of rhesus macaques. J Virol. 2011 Apr;85(8):4025-30. DOI: 10.1128/JVI.02292-10

66. Ivanova N, Sotirova Y, Gavrailov G, Nikolova K, Andonova V. Advances in the Prophylaxis of Respiratory Infections by the Nasal and the Oromucosal Route: Relevance to the Fight with the SARS-CoV-2 Pandemic. Pharmaceutics. 2022 Feb;14(3):. DOI: 10.3390/pharmaceutics14030530

67. Jiang X, Luo M, Zou Z, Wang X, Chen C, Qiu J. Asymptomatic SARS-CoV-2 infected case with viral detection positive in stool but negative in nasopharyngeal samples lasts for 42 days. J Med Virol. 2020 Oct;92(10):1807-9. DOI: 10.1002/jmv.25941

68. Zou L, Ruan F, Huang M, Liang L, Huang H, Hong Z, Yu J, Kang M, Song Y, Xia J, Guo Q, Song T, He J, Yen HL, Peiris M, Wu J. SARS-CoV-2 Viral Load in Upper Respiratory Specimens of Infected Patients. N Engl J Med. 2020 Mar;382(12):1177-9. DOI: 10.1056/NEJMc2001737

69. Stathis C, Victoria N, Loomis K, Nguyen SA, Eggers M, Septimus E, Safdar N. Review of the use of nasal and oral antiseptics during a global pandemic. Future Microbiol. 2021 Jan;16(2):119-30. DOI: 10.2217/fmb-2020-0286

70. Hamming I, Timens W, Bulthuis ML, Lely AT, Navis G, van Goor H. Tissue distribution of ACE2 protein, the functional receptor for SARS coronavirus. A first step in understanding SARS pathogenesis. J Pathol. 2004 Jun;203(2):631-7. DOI: 10.1002/path.1570

71. Mittal A, Manjunath K, Ranjan RK, Kaushik S, Kumar S, Verma V. COVID-19 pandemic: Insights into structure, function, and hACE2 receptor recognition by SARS-CoV-2. PLoS Pathog. 2020 Aug;16(8):e1008762. DOI: 10.1371/journal.ppat.1008762

72. Zhou F, Yu T, Du R, Fan G, Liu Y, Liu Z, Xiang J, Wang Y, Song B, Gu X, Guan L, Wei Y, Li H, Wu X, Xu J, Tu S, Zhang Y, Chen H, Cao B. Clinical course and risk factors for mortality of adult inpatients with COVID-19 in Wuhan, China: a retrospective cohort study. Lancet. 2020 Mar;395(10229):1054-62. DOI: 10.1016/S0140-6736(20)30566-3

73. Michelen M, Manoharan L, Elkheir N, Cheng V, Dagens A, Hastie C, O'Hara M, Suett J, Dahmash D, Bugaeva P, Rigby I, Munblit D, Harriss E, Burls A, Foote C, Scott J, Carson G, Olliaro P, Sigfrid L, Stavropoulou C. Characterising long COVID: a living systematic review. BMJ Glob Health. 2021 Sep;6(9):. DOI: 10.1136/bmjgh-2021-005427

74. Hawkins CL, Davies MJ. Hypochlorite-induced damage to DNA, RNA, and polynucleotides: formation of chloramines and nitrogen-centered radicals. Chem Res Toxicol. 2002 Jan;15(1):83-92. DOI: 10.1021/tx015548d

75. Stubbs AD, Lao M, Wang C, Abbatt JPD, Hoffnagle J, VandenBoer TC, Kahan TF. Near-source hypochlorous acid emissions from indoor bleach cleaning. Environ Sci Process Impacts. 2023 Jan 25;25(1):56-65. DOI: 10.1039/d2em00405d

76. Sagripanti JL, Bonifacino A. Cytotoxicity of liquid disinfectants. Surg Infect (Larchmt).

2000;1(1):3-14. DOI: 10.1089/109629600321245

77. Rai NK, Ashok A, Akondi BR. Consequences of chemical impact of disinfectants: safe preventive measures against COVID-19. Crit Rev Toxicol. 2020 Jul;50(6):513-20. DOI: 10.1080/10408444.2020.1790499

78. Mohapatra A, Wexler P. Chapter 68 – Web-Based Databases. In: Wexler P, Gilbert SG, Hakkinen PJ, Mohapatra A, editors. Information Resources in Toxicology (Fourth Edition). San Diego: Academic Press; 2009. p. 619–30 DOI: 10.1016/B978-0-12-373593-5.00068-9

79. Pullar JM, Winterbourn CC, Vissers MC. Loss of GSH and thiol enzymes in endothelial cells exposed to sublethal concentrations of hypochlorous acid. Am J Physiol. 1999 Oct;277(4):H1505-12. DOI: 10.1152/ajpheart.1999.277.4.H1505

80. Hatanaka N, Yasugi M, Sato T, Mukamoto M, Yamasaki S. Hypochlorous acid solution is a potent antiviral agent against SARS-CoV-2. J Appl Microbiol. 2022 Feb;132(2):1496-502. DOI: 10.1111/jam.15284

81. Heggers JP, Sazy JA, Stenberg BD, Strock LL, McCauley RL, Herndon DN, Robson MC. Bactericidal and wound-healing properties of sodium hypochlorite solutions: the 1991 Lindberg Award. J Burn Care Rehabil. 1991;12(5):420-4. DOI: 10.1097/00004630-199109000-00005

82. Reis R, Sipahi H, Dinc O, Kavaz T, Charehsaz M, Dimoglo A, Aydın A. Toxicity, mutagenicity and stability assessment of simply produced electrolyzed water as a wound healing agent in vitro. Hum Exp Toxicol. 2021 Mar;40(3):452-63. DOI: 10.1177/0960327120952151

83. Goh CF, Ming LC, Wong LC. Dermatologic reactions to disinfectant use during the COVID-19 pandemic. Clin Dermatol. 2021;39(2):314-22. DOI: 10.1016/j.clindermatol.2020.09.005

84. EPA. Distributed Structure-Searchable Toxicity (DSSTox) Database. Durham, NC: EPA; 2023 Jan 06 [last access 2023 Feb 10]. Available from: https://www.epa.gov/chemical-research/distributed-structure-searchable-toxicity-dsstox-database

85. Agency for Toxic Substances and Disease Registry. [last access 2023 Feb 10]. Available from: https://www.atsdr.cdc.gov/

86. European Commission. “Regulation (EU) No 528/2012 concerning the making available on the market and use of biocidal products”, Slovak Republic. 2020.

87. Breves R. Testbericht 20-14998-1 über die bakterizide Wirksamkeit von Biodyozon – vernebelt mit oji.live mittels adhoc entwickelter Hausmethode zur Darstellung der mikrobiziden Wirksamkeit Biozid-haltiger Aerosole auf als Aerosol vernebelte Mikroorganismen [unpublished]. Düsseldorf: Henkel Corporate Scientific Services/Microbiology; 2020.

88. Rasmussen ED, Williams JF. Stabilized hypochlorous acid disinfection for highly vulnerable populations: Brio HOCL™ wound disinfection and area decontamination. In: 2017 IEEE Global Humanitarian Technology Conference (GHTC); 2017. p. 1-8. DOI: 10.1109/GHTC.2017.8239259

89. Boecker D, Breves R, Bulitta C, Zhang Z. Antimicrobial activity in the gasphase with hypochloric acid. In: Abstracts 6th Int Conf Prevention Infection Control (ICPIC). Antimicrobial Resist Infect Contr. 2021;10(Suppl 1):97. DOI: 10.1186/s13756-021-00974-z

90. Boecker D, Breves R, Zhang Z, Bulitta C. Antimicrobial Activity in the Gasphase with Hypochloric Acid. Current Directions in Biomedical Engineering. 2021;7(2): 511-4. DOI: 10.1515/cdbme-2021-2130

91. Burd JF. Ten-day exposure to hypochlorous acid (wonder spray) fog results in no detectable effect on blood metabolic panel and minimal lung pathology. J Pulm Med

2020;4(5):1-3.

92. Yu MS, Kim BH, Kang SH, Lim DJ. Low-concentration hypochlorous acid nasal irrigation for chronic sinonasal symptoms: a prospective randomized placebo-controlled study. Eur Arch Otorhinolaryngol. 2017 Mar;274(3):1527-33. DOI: 10.1007/s00405-016-4387-5

93. Cho HJ, Min HJ, Chung HJ, Park DY, Seong SY, Yoon JH, Lee JG, Kim CH. Improved outcomes after low-concentration hypochlorous acid nasal irrigation in pediatric chronic sinusitis. Laryngoscope. 2016 Apr;126(4):791-5. DOI: 10.1002/lary.25605

94. Boecker D, Breves R, Bulitta C, Herth F, Zhang Z. Safe, Effective, and cost-efficient air cleaning for populated rooms and entire buildings based on the disinfecting power of vaporized hypochlorous acid. Int J Health Med Engineer. 2022;16(12):209-13.

95. Font-Ribera L, Kogevinas M, Zock JP, Gómez FP, Barreiro E, Nieuwenhuijsen MJ, Fernandez P, Lourencetti C, Pérez-Olabarría M, Bustamante M, Marcos R, Grimalt JO, Villanueva CM. Short-term changes in respiratory biomarkers after swimming in a chlorinated pool. Environ Health Perspect. 2010 Nov;118(11):1538-44. DOI: 10.1289/ehp.1001961

96. Richardson SD, DeMarini DM, Kogevinas M, Fernandez P, Marco E, Lourencetti C, Ballesté C, Heederik D, Meliefste K, McKague AB, Marcos R, Font-Ribera L, Grimalt JO, Villanueva CM. What's in the pool? A comprehensive identification of disinfection by-products and assessment of mutagenicity of chlorinated and brominated swimming pool water. Environ Health Perspect. 2010 Nov;118(11):1523-30. DOI: 10.1289/ehp.1001965

97. Gomà A, de Lluis R, Roca-Ferrer J, Lafuente J, Picado C. Respiratory, ocular and skin health in recreational and competitive swimmers: Beneficial effect of a new method to reduce chlorine oxidant derivatives. Environ Res. 2017 Jan;152:315-21. DOI: 10.1016/j.envres.2016.10.030

98. Font-Ribera L, Kogevinas M, Zock JP, Gómez FP, Barreiro E, Nieuwenhuijsen MJ, Fernandez P, Lourencetti C, Pérez-Olabarría M, Bustamante M, Marcos R, Grimalt JO, Villanueva CM. Short-term changes in respiratory biomarkers after swimming in a chlorinated pool. Environ Health Perspect. 2010 Nov;118(11):1538-44. DOI: 10.1289/ehp.1001961

99. Catto C, Sabrina S, Ginette CT, Manuel R, Robert T. Occurrence and spatial and temporal variations of disinfection by-products in the water and air of two indoor swimming pools. Int J Environ Res Public Health. 2012 Aug;9(8):2562-86. DOI: 10.3390/ijerph9082562

100. Foncubierta Blázquez JL, Maestre IR, González Gallero FJ, Álvarez Gómez P. Experimental test for the estimation of the evaporation rate in indoor swimming pools: Validation of a new CFD-based simulation methodology. Build Environ. 2018;138:293-9. DOI: 10.1016/J.BUILDENV.2018.05.008.

101. Judd SJ, Bullock G. The fate of chlorine and organic materials in swimming pools. Chemosphere. 2003 Jun;51(9):869-79. DOI: 10.1016/S0045-6535(03)00156-5

102. Weng SC, Weaver WA, Afifi MZ, Blatchley TN, Cramer JS, Chen J, Blatchley ER 3rd. Dynamics of gas-phase trichloramine (NCl3) in chlorinated, indoor swimming pool facilities. Indoor Air. 2011 Oct;21(5):391-9. DOI: 10.1111/j.1600-0668.2011.00710.x

103. DGUV. Forschungsprojekt Trichloramin in Bädern. Berlin: Deutsche Gesetzliche Unfallversicherung (DGUV); 2009. https://www.dguv.de/medien/inhalt/praevention/themen_a_z/gefahrstoffe/forschung/trichloramin101778.pdf

104. Basden K. Swimming pool and spa pool atmospheres. Clean Air Environ Qual 2006; 40:32–5.

105. Hsu HT, Chen MJ, Lin CH, Chou WS, Chen JH. Chloroform in indoor swimming-pool air: monitoring and modeling coupled with the effects of environmental conditions and occupant activities. Water Res. 2009 Aug;43(15):3693-704. DOI: 10.1016/j.watres.2009.05.032

106. Tsamba L, Correc O, Couzinet A. +Chlorination by-products in indoor swimming pools: Development of a pilot pool unit and impact of operating parameters. Environ Int. 2020 Apr;137:105566. DOI: 10.1016/j.envint.2020.105566

107. Chu H, Nieuwenhuijsen MJ. Distribution and determinants of trihalomethane concentrations in indoor swimming pools. Occup Environ Med. 2002 Apr;59(4):243-7. DOI: 10.1136/oem.59.4.243

108. Casciaro M, Di Salvo E, Pace E, Ventura-Spagnolo E, Navarra M, Gangemi S. Chlorinative stress in age-related diseases: a literature review. Immun Ageing. 2017;14:21. DOI: 10.1186/s12979-017-0104-5

109. Cook NL, Viola HM, Sharov VS, Hool LC, Schöneich C, Davies MJ. Myeloperoxidase-derived oxidants inhibit sarco/endoplasmic reticulum Ca2+-ATPase activity and perturb Ca2+ homeostasis in human coronary artery endothelial cells. Free Radic Biol Med. 2012 Mar;52(5):951-61. DOI: 10.1016/j.freeradbiomed.2011.12.001

110. Prütz WA. Hypochlorous acid interactions with thiols, nucleotides, DNA, and other biological substrates. Arch Biochem Biophys. 1996 Aug;332(1):110-20. DOI: 10.1006/abbi.1996.0322

111. Pattison DI, Hawkins CL, Davies MJ. Hypochlorous acid-mediated oxidation of lipid components and antioxidants present in low-density lipoproteins: absolute rate constants, product analysis, and computational modeling. Chem Res Toxicol. 2003 Apr;16(4):439-49. DOI: 10.1021/tx025670s

112. Pattison DI, Davies MJ. Absolute rate constants for the reaction of hypochlorous acid with protein side chains and peptide bonds. Chem Res Toxicol. 2001 Oct;14(10):1453-64. DOI: 10.1021/tx0155451

113. Armesto XL, Canle L. M, Fernández MI, García M v., Santaballa JA. First steps in the oxidation of sulfur-containing amino acids by hypohalogenation: very fast generation of intermediate sulfenyl halides and halosulfonium cations. Tetrahedron. 2000;56(8):1103-9. DOI: 10.1016/S0040-4020(99)01066-2

114. Armesto XL, Canle LM, García MV, Santaballa JA. Aqueous chemistry of N-halo-compounds. Chem Soc Rev. 1998;27(6):453-60. DOI: 10.1039/A827453Z

115. Hawkins CL. Hypochlorous acid-mediated modification of proteins and its consequences. Essays Biochem. 2020 Feb;64(1):75-86. DOI: 10.1042/EBC20190045

116. Dumas O, Varraso R, Boggs KM, Quinot C, Zock JP, Henneberger PK, Speizer FE, Le Moual N, Camargo CA Jr. Association of Occupational Exposure to Disinfectants With Incidence of Chronic Obstructive Pulmonary Disease Among US Female Nurses. JAMA Netw Open. 2019 Oct;2(10):e1913563. DOI: 10.1001/jamanetworkopen.2019.13563

117. Collins DB, Farmer DK. Unintended Consequences of Air Cleaning Chemistry. Environ Sci Technol. 2021 Sep;55(18):12172-9. DOI: 10.1021/acs.est.1c02582

118. Collins D. The (Air) Chemistry Behind Fogging for SARS-CoV-2 Disinfection. 2020 [last access 2023 Feb 10]. Available from: https://dbc007.medium.com/the-air-chemistry-behind-fogging-for-sars-cov-2-disinfection-ac3df05326bc

119. Soffritti M, Belpoggi F, Lenzi A, Maltoni C. Results of long-term carcinogenicity studies of chlorine in rats. Ann N Y Acad Sci. 1997 Dec;837:189-208. DOI: 10.1111/j.1749-6632.1997.tb56875.x

120. Kurokawa Y, Takayama S, Konishi Y, Hiasa Y, Asahina S, Takahashi M, Maekawa

A, Hayashi Y. Long-term in vivo carcinogenicity tests of potassium bromate, sodium hypochlorite, and sodium chlorite conducted in Japan. Environ Health Perspect. 1986 Nov;69:221-35. DOI: 10.1289/ehp.8669221

121. Abdel-Rahman MS, Berardi MR, Bull RJ. Effect of chlorine and monochloramine in drinking water on the developing rat fetus. J Appl Toxicol. 1982;2(3):156-9. DOI: 10.1002/jat.2550020307

122. Sassa A, Kamoshita N, Matsuda T, Ishii Y, Kuraoka I, Nohmi T, Ohta T, Honma M, Yasui M. Miscoding properties of 8-chloro-2'-deoxyguanosine, a hypochlorous acid-induced DNA adduct, catalysed by human DNA polymerases. Mutagenesis. 2013 Jan;28(1):81-8. DOI: 10.1093/mutage/ges056

123. Finewax Z, Pagonis D, Claflin MS, Handschy AV, Brown WL, Jenks O, Nault BA, Day DA, Lerner BM, Jimenez JL, Ziemann PJ, de Gouw JA. Quantification and source characterization of volatile organic compounds from exercising and application of chlorine-based cleaning products in a university athletic center. Indoor Air. 2021 Sep;31(5):1323-39. DOI: 10.1111/ina.12781

124. Mattila JM, Lakey PSJ, Shiraiwa M, Wang C, Abbatt JPD, Arata C, Goldstein AH, Ampollini L, Katz EF, DeCarlo PF, Zhou S, Kahan TF, Cardoso-Saldaña FJ, Ruiz LH, Abeleira A, Boedicker EK, Vance ME, Farmer DK. Multiphase Chemistry Controls Inorganic Chlorinated and Nitrogenated Compounds in Indoor Air during Bleach Cleaning. Environ Sci Technol. 2020 Feb;54(3):1730-9. DOI: 10.1021/acs.est.9b05767

125. Dewey HM, Jones JM, Keating MR, Budhathoki-Uprety J. Increased use of disinfectants during the COVID-19 pandemic and its potential impacts on health and safety. ACS Chem Health Saf. 2021;29(1):27-38. DOI: 10.1021/ACS.CHAS.1C00026.

126. Weiss SJ. Tissue destruction by neutrophils. N Engl J Med. 1989 Feb;320(6):365-76. DOI: 10.1056/NEJM198902093200606

127. Eaton JW. Defenses against hypochlorous acid: parrying the neutrophil's rapier thrust. J Lab Clin Med. 1993 Feb;121(2):197-8.

128. Akong-Moore K, Chow OA, von Köckritz-Blickwede M, Nizet V. Influences of chloride and hypochlorite on neutrophil extracellular trap formation. PLoS One. 2012;7(8):e42984. DOI: 10.1371/journal.pone.0042984

129. Chapman I, Hsu JTS, Stankiewicz K, Bhatia AC. Use of Hypochlorous Acid as a Preoperative Antiseptic Before Placement of Dermal Fillers: An Alternative to the Standard Options. Dermatol Surg. 2018 Apr;44(4):597-9. DOI: 10.1097/DSS.0000000000001245

130. Stroman DW, Mintun K, Epstein AB, Brimer CM, Patel CR, Branch JD, Najafi-Tagol K. Reduction in bacterial load using hypochlorous acid hygiene solution on ocular skin. Clin Ophthalmol. 2017;11:707-14. DOI: 10.2147/OPTH.S132851

131. Agafonova E, Reshetnikova I, Rizvanova F. Mucosal Immunity After Novel COVID-19 Infection - Virus-Induced Immunosuppression: Preliminary Study. Bionanoscience. 2022;12(4):1473-81. DOI: 10.1007/s12668-022-01020-x

132. Thorn RM, Lee SW, Robinson GM, Greenman J, Reynolds DM. Electrochemically activated solutions: evidence for antimicrobial efficacy and applications in healthcare environments. Eur J Clin Microbiol Infect Dis. 2012 May;31(5):641-53. DOI: 10.1007/s10096-011-1369-9

133. Delgado-Enciso I, Paz-Garcia J, Barajas-Saucedo CE, Mokay-Ramírez KA, Meza-Robles C, Lopez-Flores R, Delgado-Machuca M, Murillo-Zamora E, Toscano-Velazquez JA, Delgado-Enciso J, Melnikov V, Walle-Guillen M, Galvan-Salazar HR, Delgado-Enciso OG, Cabrera-Licona A, Danielewicz-Mata EJ, Mandujano-Diaz PJ, Guzman-Esquivel J, Montes-Galindo DA, Perez-Martinez H, Jimenez-Villegaz JM, Hernandez-Rangel AE, Montes-Diaz P, Rodriguez-Sanchez IP, Martinez-Fierro ML,

Garza-Veloz I, Tiburcio-Jimenez D, Zaizar-Fregoso SA, Gonzalez-Alcaraz F, Gutierrez-Gutierrez L, Diaz-Lopez L, Ramirez-Flores M, Guzman-Solorzano HP, Gaytan-Sandoval G, Martinez-Perez CR, Espinoza-Gómez F, Rojas-Larios F, Hirsch-Meillon MJ, Baltazar-Rodriguez LM, Barrios-Navarro E, Oviedo-Rodriguez V, Mendoza-Hernandez MA, Prieto-Diaz-Chavez E, Paz-Michel BA. Safety and efficacy of a COVID-19 treatment with nebulized and/or intravenous neutral electrolyzed saline combined with usual medical care vs. usual medical care alone: A randomized, open-label, controlled trial. Exp Ther Med. 2021 Sep;22(3):915. DOI: 10.3892/etm.2021.10347

134. Zaizar-Fregoso SA, Paz-Michel BA, Rodriguez-Hernandez A, Paz-Garcia J, Aurelien-Cabezas NS, Tiburcio-Jimenez D, Melnikov V, Murillo-Zamora E, Delgado-Enciso OG, Cabrera-Licona A, Guzman-Esquivel J, Barajas-Saucedo CE, Rodriguez-Sanchez IP, Martinez-Fierro ML, Moy-López NA, Lara-Esqueda A, Guzman-Muñiz J, Delgado-Machuca M, Delgado-Enciso I. Systemic Administration of Neutral Electrolyzed Saline as a Novel Treatment for Rheumatoid Arthritis Reduces Mechanical and Inflammatory Damage to the Joints: Preclinical Evaluation in Mice. Evid Based Complement Alternat Med. 2022;2022:1717614. DOI: 10.1155/2022/1717614

135. Papsin B, McTavish A. Saline nasal irrigation: Its role as an adjunct treatment. Can Fam Physician. 2003 Feb;49:168-73.

136. Adam P, Stiffman M, Blake RL Jr. A clinical trial of hypertonic saline nasal spray in subjects with the common cold or rhinosinusitis. Arch Fam Med. 1998;7(1):39-43. DOI: 10.1001/archfami.7.1.39

137. Kim KC, Singh BN. Hydrophobicity of mucin-like glycoproteins secreted by cultured tracheal epithelial cells: association with lipids. Exp Lung Res. 1990;16(3):279-92. DOI: 10.3109/01902149009108845

138. Yu MS, Park HW, Kwon HJ, Jang YJ. The effect of a low concentration of hypochlorous acid on rhinovirus infection of nasal epithelial cells. Am J Rhinol Allergy. 2011;25(1):40-4. DOI: 10.2500/ajra.2011.25.3545

139. House SA, Gadomski AM, Ralston SL. Evaluating the Placebo Status of Nebulized Normal Saline in Patients With Acute Viral Bronchiolitis: A Systematic Review and Meta-analysis. JAMA Pediatr. 2020 Mar;174(3):250-9. DOI: 10.1001/jamapediatrics.2019.5195

140. Brindle CT, Porter S, Bijlani K, Arumugam S, Matias R, Najafi R, Fisher J. Preliminary Results of the Use of a Stabilized Hypochlorous Acid Solution in the Management of Ralstonia Pickettii Biofilm on Silicone Breast Implants. Aesthet Surg J. 2018 May;38(suppl_2):S52-S61. DOI: 10.1093/asj/sjx229

141. Haws MJ, Gingrass MK, Porter RS, Brindle CT. Surgical Breast Pocket Irrigation With Hypochlorous Acid (HOCl): An In Vivo Evaluation of Pocket Protein Content and Potential HOCl Antimicrobial Capacity. Aesthet Surg J. 2018 Oct;38(11):1178-84. DOI: 10.1093/asj/sjy031

142. Finger PT, Fam A, Tomar AS, Garg G, Chin KJ. COVID-19 prophylaxis in ophthalmology. Indian J Ophthalmol. 2020 Oct;68(10):2062-3. DOI: 10.4103/ijo.IJO_2459_20

143. Block MS, Rowan BG. Hypochlorous Acid: A Review. J Oral Maxillofac Surg. 2020 Sep;78(9):1461-6. DOI: 10.1016/j.joms.2020.06.029

144. Mueller CA, Winter M, Renner B. A Concept for the Reduction of Mucosal SARS-CoV-2 Load using Hypochloric Acid Solutions. Drug Res (Stuttg). 2021 Jul;71(6):348-50. DOI: 10.1055/a-1467-5956

145. Wongkietkachorn A, Surakunprapha P, Wittayapairoch J, Wongkietkachorn N, Wongkietkachorn S. The Use of Hypochlorous Acid Lavage to Treat Infected Cavity Wounds. Plast Reconstr Surg Glob Open. 2020 Jan;8(1):e2604. DOI: 10.1097/GOX.0000000000002604

146. Matthews MR, Quan AN, Weir AS, Foster KN, Caruso DM. Temporary abdominal closure combined with an irrigating system utilizing hypochlorous acid solution to decrease abdominal mucopurulence. Eplasty. 2018;18:e12.

147. Robson MC, Payne WG, Ko F, Mentis M, Donati G, Shafii SM, et al. Hypochlorous acid as a potential wound care agent part ii. stabilized hypochlorous acid: its role in decreasing tissue bacterial bioburden and overcoming the inhibition of infection on wound healing. J Burns Wounds. 2007;6:80-90.

148. Gold MH, Andriessen A, Bhatia AC, Bitter P Jr, Chilukuri S, Cohen JL, Robb CW. Topical stabilized hypochlorous acid: The future gold standard for wound care and scar management in dermatologic and plastic surgery procedures. J Cosmet Dermatol. 2020 Feb;19(2):270-7. DOI: 10.1111/jocd.13280

149. Kramer A, Dissemond J, Kim S, Willy C, Mayer D, Papke R, Tuchmann F, Assadian O. Consensus on Wound Antisepsis: Update 2018. Skin Pharmacol Physiol. 2018;31(1):28-58. DOI: 10.1159/000481545

150. Jull A, Wadham A, Bullen C, Parag V, Parsons JGM, Laking G, Waters J, Klonizakis M, O'Brien J. Prescribed exercise regimen versus usual care and hypochlorous acid wound solution versus placebo for treating venous leg ulcers: study protocol for a randomised controlled trial (Factorial4VLU). BMJ Open. 2021 Feb;11(2):e043420. DOI: 10.1136/bmjopen-2020-043420

151. Bongiovanni CM. Effects of Hypochlorous Acid Solutions on Venous Leg Ulcers (VLU): Experience With 1249 VLUs in 897 Patients. J Am Coll Clin Wound Spec. 2014 Dec;6(3):32-7. DOI: 10.1016/j.jccw.2016.01.001

152. Selkon JB, Cherry GW, Wilson JM, Hughes MA. Evaluation of hypochlorous acid washes in the treatment of chronic venous leg ulcers. J Wound Care. 2006 Jan;15(1):33-7. DOI: 10.12968/jowc.2006.15.1.26861

153. Wang Y, Graves DT. Keratinocyte Function in Normal and Diabetic Wounds and Modulation by FOXO1. J Diabetes Res. 2020;2020:3714704. DOI: 10.1155/2020/3714704

154. Chen CJ, Chen CC, Ding SJ. Effectiveness of Hypochlorous Acid to Reduce the Biofilms on Titanium Alloy Surfaces in Vitro. Int J Mol Sci. 2016 Jul;17(7):. DOI: 10.3390/ijms17071161

155. Niezgoda JA, Sordi PJ, Hermans MH. Evaluation of Vashe Wound Therapy in the clinical management of patients with chronic wounds. Adv Skin Wound Care. 2010 Aug;23(8):352-7. DOI: 10.1097/01.ASW.0000383198.35815.a2

156. Huxtable RJ. Physiological actions of taurine. Physiol Rev. 1992 Jan;72(1):101-63. DOI: 10.1152/physrev.1992.72.1.101

157. Rosso J, Neal B. Status report on topical hypochlorous acid: clinical relevance of specific formulations, potential modes of action, and study outcomes. J Clin Aesthet Dermatol. 2018;11(11):36-9.

158. Sipahi H, Reis R, Dinc O, Kavaz T, Dimoglo A, Aydın A. In vitro biocompatibility study approaches to evaluate the safety profile of electrolyzed water for skin and eye. Hum Exp Toxicol. 2019 Nov;38(11):1314-26. DOI: 10.1177/0960327119862333

159. Yan P, Daliri EB, Oh DH. New Clinical Applications of Electrolyzed Water: A Review. Microorganisms. 2021 Jan;9(1). DOI: 10.3390/microorganisms9010136

160. Armstrong DG, Bohn G, Glat P, Kavros SJ, Kirsner R, Snyder R, Tettelbach W. Expert Recommendations for the Use of Hypochlorous Solution: Science and Clinical Application. Ostomy Wound Manage. 2015 May;61(5):S2-S19.

161. Datanasio N, Capezzone De Joannon A, Mangano G, Meloni M, Giarratana N, Milanese C, et al. A new acid-oxidizing solution: assessment of its role on methicillin-resistant Staphylococcus aureus (MRSA) biofilm morphological changes. Wounds. 2015;27(10):265-73.

162. He X, Lau EHY, Wu P, Deng X, Wang J, Hao X, Lau YC, Wong JY, Guan Y, Tan X, Mo X, Chen Y, Liao B, Chen W, Hu F, Zhang Q, Zhong M, Wu Y, Zhao L, Zhang F, Cowling BJ, Li F, Leung GM. Temporal dynamics in viral shedding and transmissibility of COVID-19. Nat Med. 2020 May;26(5):672-5. DOI: 10.1038/s41591-020-0869-5

163. Kramer A, Eggers M, Exner M, Hübner NO, Simon A, Steinmann E, et al. Prävention von COVID-19 durch viruzides Gurgeln und viruziden Nasenspray - aktualisierte Fassung April 2022. Hyg Med. 2022;47:73-7.

164. Giarratana N, Rajan B, Kamala K, Mendenhall M, Reiner G. A sprayable Acid-Oxidizing solution containing hypochlorous acid (AOS2020) efficiently and safely inactivates SARS-Cov-2: a new potential solution for upper respiratory tract hygiene. Eur Arch Otorhinolaryngol. 2021 Aug;278(8):3099-103. DOI: 10.1007/s00405-021-06644-5

165. Kramer A, Eggers M. Prävention respiratorischer Virusinfektionen durch viruzide Schleimhautantiseptik bei medizinischem Personal und in der Bevölkerung. Hyg Med. 2020;45 (9):D119-26.

166. Yu MS, Kim BH, Kang SH, Lim DJ. Low-concentration hypochlorous acid nasal irrigation for chronic sinonasal symptoms: a prospective randomized placebo-controlled study. Eur Arch Otorhinolaryngol. 2017 Mar;274(3):1527-33. DOI: 10.1007/s00405-016-4387-5

167. Burd JF. Data calculation wonder spray (HOCL) kills the bacteria that cause strep throat and pneumonia. Online J Otolaryngol Rhinol. 2019;2(1). DOI: 10.33552/OJOR.2019.02.000527

168. Gutiérrez-García R, De La Cerda-Ángeles JC, Cabrera-Licona A, Delgado-Enciso I, Mervitch-Sigal N, Paz-Michel BA. Nasopharyngeal and oropharyngeal rinses with neutral electrolyzed water prevents COVID-19 in front-line health professionals: A randomized, open-label, controlled trial in a general hospital in Mexico City. Biomed Rep. 2022 Feb;16(2):11. DOI: 10.3892/br.2021.1494

169. Kim HJ, Lee JG, Kang JW, Cho HJ, Kim HS, Byeon HK, Yoon JH. Effects of a low concentration hypochlorous Acid nasal irrigation solution on bacteria, fungi, and virus. Laryngoscope. 2008 Oct;118(10):1862-7. DOI: 10.1097/MLG.0b013e31817f4d34

170. Wu SH, Lin JF, Jiang RS. Antibacterial Effect of Hypochlorous Acid Solution on Nasal Discharge from Patients with Chronic Rhinosinusitis. Int J Otolaryngol. 2018;2018:8568694. DOI: 10.1155/2018/8568694

171. Cho HJ, Min HJ, Chung HJ, Park DY, Seong SY, Yoon JH, Lee JG, Kim CH. Improved outcomes after low-concentration hypochlorous acid nasal irrigation in pediatric chronic sinusitis. Laryngoscope. 2016 Apr;126(4):791-5. DOI: 10.1002/lary.25605

172. Yu MS, Park HW, Kwon HJ, Jang YJ. The effect of a low concentration of hypochlorous acid on rhinovirus infection of nasal epithelial cells. Am J Rhinol Allergy. 2011;25(1):40-4. DOI: 10.2500/ajra.2011.25.3545

173. Esteripharma. ESTERIflu® Antiséptico Nasal [last access 2023 Feb 10]. Available from: https://esteriflu.com/

174. Galfetti P. Klinische Studie mit neuartigem Nasenspray mit dem Codenamen APR-AOS2020 bei Patienten mit leichter COVID-19 eingeleitet. 2021 [last access 2023 Feb 10]. Available from: https://www.ots.at/presseaussendung/OTS_20210513_OTS0054/klinische-studie-mit-neuartigem-nasenspray-mit-dem-codenamen-apr-aos2020-bei-patienten-mit-leichter-covid-19-eingeleitet

175. Mohamed NA, Baharom N, Shahida W, Sulaiman W, Rashid ZZ, Kon Ken W, et al. Early viral clearance among covid-19 patients when gargling with povidone-iodine

and essential oils – a clinical trial [Preprint]. medRxiv. 2020. DOI: 10.1101/2020.09.07.20180448

176. Davies K, Buczkowski H, Welch SR, Green N, Mawer D, Woodford N, Roberts ADG, Nixon PJ, Seymour DW, Killip MJ. Effective in vitro inactivation of SARS-CoV-2 by commercially available mouthwashes. J Gen Virol. 2021 Apr;102(4):001578. DOI: 10.1099/jgv.0.001578

177. Mezarina Mendoza JPI, Trelles Ubillús BP, Salcedo Bolívar GT, Castañeda Palacios RDP, Herrera Lopez PSG, Padilla Rodríguez DA, Uchima Koecklin KH. Antiviral effect of mouthwashes against SARS-COV-2: A systematic review. Saudi Dent J. 2022 Mar;34(3):167-93. DOI: 10.1016/j.sdentj.2022.01.006

178. Küster I, Kramer A, Bremert T, Langner S, Hosemann W, Beule AG. Eradication of MRSA skull base osteitis by combined treatment with antibiotics and sinonasal irrigation with sodium hypochlorite. Eur Arch Otorhinolaryngol. 2016 Jul;273(7):1951-6. DOI: 10.1007/s00405-015-3739-x

179. Morita C, Nishida T, Ito K. Biological toxicity of acid electrolyzed functional water: effect of oral administration on mouse digestive tract and changes in body weight. Arch Oral Biol. 2011 Apr;56(4):359-66. DOI: 10.1016/j.archoralbio.2010.10.016

180. Watson RD. Oral administration of electrolyzed water for treatment and prevention of PEDv in swine, swine herds and swine confinements. US 9,283,250 B2, 2014.

181. House SA, Gadomski AM, Ralston SL. Evaluating the Placebo Status of Nebulized Normal Saline in Patients With Acute Viral Bronchiolitis: A Systematic Review and Meta-analysis. JAMA Pediatr. 2020 Mar;174(3):250-9. DOI: 10.1001/jamapediatrics.2019.5195

182. Ludwig M, Enzenhofer E, Schneider S, Rauch M, Bodenteich A, Neumann K, Prieschl-Grassauer E, Grassauer A, Lion T, Mueller CA. Efficacy of a carrageenan nasal spray in patients with common cold: a randomized controlled trial. Respir Res. 2013 Nov;14(1):124. DOI: 10.1186/1465-9921-14-124

183. European Commission. Active chlorine generated from sodium chloride by electrolysis. Regulation (EU) No 528/2012. 2020;(68):48-119.

184. Bowe B, Xie Y, Al-Aly Z. Acute and postacute sequelae associated with SARS-CoV-2 reinfection. Nat Med. 2022 Nov;28(11):2398-405. DOI: 10.1038/s41591-022-02051-3

185. Luo B, Schaub A, Glas I, Klein LK, David SC, Bluvshtein N, Violaki K, Motos G, Pohl MO, Hugentobler W, Nenes A, Krieger UK, Stertz S, Peter T, Kohn T. Expiratory Aerosol pH: The Overlooked Driver of Airborne Virus Inactivation. Environ Sci Technol. 2023 Jan;57(1):486-97. DOI: 10.1021/acs.est.2c05777

186. Henkens M. A.18 – Hypochlorous acid (HOCl). Geneva: Medecins sans Frontieres; 2021. Available from: https://cdn.who.int/media/docs/default-source/essential-medicines/2021-eml-expert-committee/public-comments/a18_hocl_msf.pdf?sfvrsn=db678ad2_5

187. ECHA; Biocidal Products Committee (BPC). Opinion on the application for approval of the active substance: Active chlorine released from hypochlorous acid Product type: 2. Helsinki: ECHA; 2020 Jun 16. Available from: https://echa.europa.eu/documents/10162/2918787b-45e0-d084-b770-f25837cc5775

188. World Health Organization. Cleaning and disinfection of environmental surfaces in the context of COVID-19: interim guidance, 15 May 2020. Geneva: World Health Organization; 2020 [last access 2023 Feb 10]. Available from: https://apps.who.int/iris/handle/10665/332096

189. EPA. About List N: Disinfectants for Coronavirus (COVID-19). Washington, DC: U.S. Environmental Protection Agency; 2022 May 22 [last access 2023 Feb 10]. Available from: https://www.epa.gov/coronavirus/about-list-n-disinfectants-coronavirus-covid-19-0

190. FDA. Environmental Assessment for Food Contact Notification FCN 1811. 2017. Available from: https://www.fda.gov/files/food/published/Environmental-Assessment-for-Food-Contact-Notification-No.-1811.pdf

191. FDA. Environmental decision memo for food contact notification No.1811. 2017 [last access 2023 Feb 10]. Available from: https://www.fda.gov/food/environmental-decisions/environmental-decision-memo-food-contact-notification-no-1811

192. FDA. Inventory of effective food contact substance (FCS) Notifications. 2022 [last access 2023 Feb 10]. Available from: https://www.cfsanappsexternal.fda.gov/scripts/fdcc/?set=FCN

193. Government of Canada. Hard-surface disinfectants and hand sanitizers (COVID-19): List of disinfectants with evidence for use against COVID-19. 2023 [last access 2023 Feb 10]. Available from:https://www.canada.ca/en/health-canada/services/drugs-health-products/disinfectants/covid-19/list.html

194. Australian Government. Disinfectants, sterilants and sanitary products. Woden: TGA; 2022 Oct 19 [last access 2023 Feb 10]. Available from: https://www.tga.gov.au/resources/resource/guidance/disinfectants-sterilants-and-sanitary-products

195. ECHA. ECHA recommends approval of three active substances. Helsinki: ECHA; 2020. pp. 1-3. Available from: https://echa.europa.eu/-/biocides-committee-recommends-approving-three-active-substances

196. ECHA. Regulation (EU) No 528/2012 concerning the making available on the market and use of biocidal products - Active chlorine released from calcium hypochlorite. Helsinki: ECHA; 2017. Available from: https://echa.europa.eu/documents/10162/38bc36da-c98c-0916-7101-c3c7299ac4ef

197. Mitsuboshi S, Yamaguchi R, Uchida H, Kamoshida S, Hashi H. Inappropriate sales of hypochlorous acid solution in Japan: An online investigation. Infect Control Hosp Epidemiol. 2021 Oct;42(10):1297-9. DOI: 10.1017/ice.2020.1248

198. BAuA. Ist eine Raumluftdesinfektion von Produktionsstätten oder anderen geschlossenen Räumen zur Inaktivierung von SARS-CoV-2-Viren durch Vernebelung von Desinfektionsmitteln (‘Kalt¬vernebelung’), z. B. Wasserstoff¬peroxid, bei laufendem Betrieb in Anwesenheit von Beschäftigten zulässig? FAQ-Nr.: 0033. Dortmund: Bundesanstalt für Arbeitsschutz und Arbeitsmedizin; 2020 Nov 10 [last access 2023 Feb 10]. Available from: https://www.baua.de/DE/Themen/Arbeitsgestaltung-im-Betrieb/Coronavirus/FAQ/FAQ-33.html

199. HGI Industries. ODOROX Hydroxyl generator, [last access 2023 Feb 10]. Available from: https://odoroxbc.com/

200. Faxon CB, Allen DT, Faxon CB, Allen DT. Chlorine chemistry in urban atmospheres: a review. Environm Chem. 2013;10(3):221-33. DOI: 10.1071/EN13026.

201. Wong JPS, Carslaw N, Zhao R, Zhou S, Abbatt JPD. Observations and impacts of bleach washing on indoor chlorine chemistry. Indoor Air. 2017 Nov;27(6):1082-90. DOI: 10.1111/ina.12402

202. Hippeli S, Elstner E. OH-Radical-Type Reactive Oxygen Species: A Short Review on the Mechanisms of OH-Radical-and Peroxynitrite Toxicity. Zeitschrift für Naturforschung C. 1997;52(9-10): 555-63. DOI: 10.1515/znc-1997-9-1001

203. Stone D, Whalley LK, Heard DE. Tropospheric OH and HO2 radicals: field measurements and model comparisons. Chem Soc Rev. 2012 Oct;41(19):6348-404. DOI: 10.1039/c2cs35140d

204. Crosley DR, Araps CJ, Doyle-Eisele M, McDonald JD. Gas-phase photolytic production of hydroxyl radicals in an ultraviolet purifier for air and surfaces. J Air Waste Manag Assoc. 2017 Feb;67(2):231-40. DOI: 10.1080/10962247.2016.1229236

205. Crosley DR. The Measurement of OH and HO2 in the Atmosphere. Journal of Atmospheric Sciences 1995; 52(19):3299-314. DOI: 10.1175/1520-0469(1995)052<3299:TMOOAH>2.0.CO;2

206. Atkinson R. Kinetics and mechanisms of the gas-phase reactions of the hydroxyl radical with organic compounds under atmospheric conditions. Chem Rev. 1985;85: 69-201. DOI: 10.1021/CR00071A004

207. HGI Industries Incorporated. Decomposition of Volatile Organic Compounds by Atmospheric Hydroxyl Free Radicals: Understanding the ‘Cascade’ Effect. 2021 [last access 2023 Feb 10]. Available from: https://rightair.com.au/decomposition-of-volatile-organic-compounds-by-atmospheric-hydroxyl-free-radicals-understanding-the-cascade-effect/

208. Araps C. Pyure Safety. 2022 [last access 2023 Feb 10]. Available from: https://vikand.com/pyure/

209. Lash HD. Hydroxyl and Air Purification. Comtech Research LLC Newsletter. 2003;2.

210. Balarashti JD, Tuttle RS. Determination of the ODOROX® MDU/RxTM System’s Efficacy against various Bioaerosols. Available from: https://pyure.com/wp-content/uploads/2021/03/ARE_Labs_ODOROX_Efficacy_vs_Bioaerosols.pdf

211. Du T, Adeleye AS, Zhang T, Yang N, Hao R, Li Y, et al. Effects of ozone and produced hydroxyl radicals on the transformation of graphene oxide in aqueous media. Environ Sci Nano. 2019;6:2484. DOI: 10.1039/c9en00361d

212. WT International. Hypochlorous acid. 2022 [last access 2023 Feb 10]. Available from: https://www.wtinternational.ma/en/hypochlorous-acid/

Attachment to: Boecker D, Zhang Z, Breves R, Herth F, Kramer A, Bulitta C. Antimicrobial efficacy, mode of action and in vivo use of hypochlorous acid (HOCl) for prevention or therapeutic support of infections. GMS Hyg Infect Control. 2023;18:Doc07. DOI: 10.3205/dgkh000433
